# Genome-Wide Association Study of Rice Diversity Panel Reveals New QTLs for Tolerance to Water Deficit Under the Egyptian Conditions

**DOI:** 10.1186/s12284-024-00703-1

**Published:** 2024-04-23

**Authors:** Mohamed I. Ghazy, Sabry A. EL-Naem, Ahmed G. Hefeina, Ahmed Sallam, Shamseldeen Eltaher

**Affiliations:** 1 Rice Research and Training Department, Field Crops Research Institute, Agricultural Research Center, Giza, 12619 Egypt; 2 Department of Plant Biotechnology, Genetic Engineering and Biotechnology Research Institute (GEBRI), University of Sadat City (USC), Sadat City, 32897 Egypt; 3 Department of Genetics, Faculty of Agriculture, Assiut University, Assiut, 71526 Egypt

**Keywords:** Rice, Water deficit, Drought, GWAS, Breeding

## Abstract

Drought has a significant impact on rice yield by restricting the crop's ability to grow and develop. Producing rice cultivars adapted to water deficit conditions is still the main interest of rice breeders and geneticists. To address this challenge, a set of 413 highly diverse rice populations were evaluated under normal and water deficit conditions for two growing seasons of 2021 and 2022. High genetic variation was found among genotypes for all studied traits. The heritability estimates ranged from 0.82 (panicle length) to 0.95 (plant height). Sterility percentage (SET%) was the most trait affected by water deficit in two growing seasons. 22 Rice genotypes were classified as drought tolerant in both years. Genome-wide association mapping was performed for all traits in the two growing seasons under both conditions using a total of 700,000 SNPs. The GWAS results revealed important and major SNPs associated with all traits. 26 Significant SNPs with stable allele effects were found to be associated with yield traits under water deficit conditions in both years. The results of this study provided rice genotypes that can be adapted under water deficit conditions and important stable SNP markers that can be used for marker-assisted selection after validation in different genetic backgrounds.

**Supplementary Information:**

The online version contains supplementary material available at 10.1186/s12284-024-00703-1.

## Introduction

Rice (*Oryza sativa* L.) is one of the main food crops and a significant contributor to global economic growth. It is the primary crop for almost two-thirds of the world's population, ranking in third place globally after maize and wheat in terms of production (Bhandari et al. 2021; Ghazy et al. 2021). Around the world, it is grown as an annual crop in a variety of climates, including tropical, subtropical, semiarid, and temperate regions. In order for rice to grow and develop optimally and produce good yields, it needs enough water throughout its entire life cycle, including standing water or constant floods (Jagadish et al. 2012). Along with being a staple food, rice is also used as a fuel source in the soap industry, as well as a significant component of livestock feed and cottage industries (rice straw) (Bhattacharyya et al. 2020; Pode 2016). To decrease rice crop yield losses in rainfed lowland areas and increase overall rice production, new rice cultivars with improved drought tolerance are required (Kumar et al. 2021; Manickavelu et al. 2006). The agro-climatic conditions have an impact on the cultivation of rice in all the world's rice-growing regions, and crop improvement has historically been considered as a crucial factor in the selection and evolution of improved rice varieties. Soil salinity and water availability for growing rice are two of the most significant agro-climatic conditions that limit yield, even for the advanced rice cultivars (Bhattacharyya et al. 2020; Wang et al. 2023; Zampieri et al. 2017).

Rice is one of the most water-intensive crops in Egypt, and the only irrigation option is constant flooding. During the summer, rice takes up around 22% of the total cultivated land in Egypt and uses 20% of the water supply. The total water requirements for the rice crop are a challenge because Egypt has limited water resources and a growing population (Abd Allah et al. 2010). The lack of irrigation water throughout various growth stages in some rice-growing areas, particularly those at the end of canal terminals in the northern Nile Delta, has been identified as one of the primary challenges to Egypt's rice production. To solve this issue, we need to figure out how to make irrigation water more productive and how to preserve irrigation water more effectively (Abd Allah et al. 2010). Utilizing short duration varieties is one of the key strategies to achieve this. Finding techniques to save more water without significantly reducing yield is essential. The development of drought-tolerant plant varieties to be grown in the regions affected by the irrigation water shortage in order to lower the overall water requirements is the second strategy for conserving irrigation water. Drought tolerance is a complex quantitative trait that is regulated by many genes because it involves a variety of adaptive physiological and biochemical processes at both the cellular and plant levels with varying effects at different stages of development, such as the seedling, vegetative, or reproductive stages(Hoang et al. 2019; Lang & Buu 2008; Yue et al. 2006). Drought is especially harmful during the reproductive stage and frequently results in a decrease in production (Hoang et al. 2019; Todaka et al. 2015; Yue et al. 2006). When drought stress occurred during the flowering stage, a spikelet sterility of 73% was noted (Hoang et al. 2019). While an extensive drought during the grain filling stage decreased grain yield by 75%, one that occurred during the vegetative stage had a minor impact on later plant development, with yield reductions of up to 30% (Bhandari et al. 2021; Wang et al. 2023).

Future genetic advancements in rice productivity will be made possible by adopting an integrative strategy that combines agronomic management techniques with plant breeding, physiological dissection of tolerant traits, and molecular genetic/genomic technology. Alternative to traditional mapping approach, selective genotyping was successfully utilized in rice breeding to map major quantitative trait loci (QTLs) for different traits under stress (Beena et al. 2013, 2018; Kimura & Imamoto 2014; Mofatto et al. 2016; Oladosu et al. 2019; Shakiba et al. 2017; N. Wang et al. 2023). Most of these quantitative trait loci (QTLs) have lowered genomic resolution and limited the availability of allelic diversity for positional cloning processes. They were discovered utilizing bi-parental or multi-parental populations (Beena et al., 2022; Bhattacharyya et al., 2020; Hoang et al. 2019; Korte & Farlow 2013; Sallam et al. 2022, 2023; Swamy et al. 2017). More recently, genome-wide association studies (GWAS) have made it possible to identify QTLs more precisely and to study the tremendous allelic variability present in natural populations (Beena et al. 2021; Hoang et al. 2019; Sallam et al. 2022, 2023). GWAS provides an effective technique and strategy for researching the genetic basis of rice drought resistance and discovering possible drought tolerance genes (Bhandari et al. 2020; Li et al. 2017). To find genetic variation that improves rice's drought tolerance, GWAS based on deep sequencing is helpful. In recent years, a number of genes for rice drought resistance have been cloned and studied, including OsMYB6 (Tang et al. 2019), DROT1 (Sun et al. 2022), and OsRINGzf1 (Chen et al. 2022), winch have shows favorable affects in regulating rice drought tolerance. They haven't, however, been used to create new rice cultivars that can withstand drought (Yi et al. 2023). The Rice Diversity Panel 1 (RDP1) is a global collection of more than 400 rice accessions that reflect the five main subpopulations found in the INWDCA and JAPONICA varietal groups. The RDP1 previously genotyped with 36,901 high quality SNPs. Recently, the RDP1 collection was genotyped with 700,000 SNP markers using a high density rice array (HDRA) (McCouch et al. 2016). The objectives of this study were to (i) evaluate the RDP1 accessions for drought tolerance at reproductive stages under Egyptian agriculture condiations (ii) conduct GWA mapping using the HDRA SNP genotypes, and the suite of bioinformatics tools developed for the RDP1, and (iii) identify SNPs and underlying candidate genes associated with tolerance to drought stress in rice at these critical developmental stages.

## Materials and Methods

### Plant Materials

The Rice Diversity Panel is formed up of 413 Asian rice (*O. sativa* L) cultivars, many of which are landraces, that come from 82 various countries. The tested material covers all the major rice-growing regions of the world. The panel contains 87 indica, 57 aus, 96 temperate japonica, 97 tropical japonica, 14 group/aromatic, and 62 highly admixed accessions. Out of 413 Asian rice, 392 were successfully growing under the Egyptian conditions. The rice diversity panel information is summarized Additional file 1: Table S1.

### Experimental Site and Drought Treatment

Field experiments were conducted at the experimental farm of Sakha Agricultural Research Station, Kafr El-Sheikh Governorate, Egypt (31° 08’ N latitude, 30° 58’ E Longitude), during 2021 and 2022 growing seasons to under normal (N) and water deficit (WD) conditions. All experiments were preceded by a Flax crop (*Linum* usitatissimum L.). The soil properties of the experimental site are presented in Table 1. The seeds of each genotype were sown in the nursery on 5 and 3 of May in the 2021 and 2022 seasons, respectively, and then transplanted to the field after 30 days. The seedlings of each genotype were individually transplanted in one row per replicate. Each row was 5.0 m long with a spacing of 20 × 20 cm among rows and hills, which was repeated three.. The well-watered condition (Normal) was performed using continuous flooding every 4 days with an adequate depth of submersion that ensured all surface areas were covered by water in each irrigation incident. The water-deficit treatment was imposed by using flush irrigation (flush irrigation is one of the surface irrigations without standing water after irrigation) every 12 days to reach the soil moisture content to the filed capacity. The stress condition was applied after 15 days from the transplantation date until maturity.

**Table 1:** Mechanical and chemical analysis of the experimental soil during the two seasons

Soil analysis	2021	2022
*Chemical analysis*		
PH	8.20	8.30
EC (dS m^−1^)	2.11	2.18
Organic matter (%)	1.55	1.65
*Soluble Cations, meq/lit.*		
Ca^++^	7.40	7.20
Mg^++^	2.85	2.65
Na^+^	13.70	14.10
K^+^	1.16	1.21
*Soluble Anions, meq/lit.*		
Co_3_^− −^	–	–
Hco_3_^−^	5.15	5.40
So_4_^− −^	7.95	8.18
Cl^−^	12.00	11.55
*Physical analysis*		
Sand (%)	13.28	13.46
Clay (%)	56.05	55.55
Silt (%)	320.67	31.04
CaCo_3_	3.85	3.35
Soil texture	Clay	Clay

Nitrogen fertilizer at a rate of 165 kg N ha^−1^ was applied in three splits in the form of urea (46.0% N). Phosphorous was applied at a rate of 37 kg P_2_O_5_ ha^−1^ as super-phosphate (15% P_2_O_5_), and potassium at a rate of 50 kg K_2_O kg/ha as potassium sulfate (48% K_2_O). Zinc fertilizer was applied at a rate of 24 kg/ha ZnSO_4_. Other standard agricultural practices such as weed control and disease protection were applied.

### Phenotypic Measurements

Under each condition, the following traits were recorded after complete heading. Five plants were taken randomly from each genotype to determine the agronomic, yield and its components characters. At ripening stage each plant was harvested individually. The data of two seasons had been shown and statistically analysis as average for both seasons.

1. Number of days to 50% heading (NDH): it was determined as number of days from date of sowing to the date of 50% heading for each treatment.
2. Flag leaf area (FLA cm^2^): the leaf area of 20 flag leaves were measured using leaf area meter (Model LI-3000A), and then the mean value of flag leaf area was calculated.
3. Plant height (PH cm): average plant height at heading stage was estimated from the soil surface to the tip of the main panicle.
4. Number of panicles per plant (NPP): the number of panicles from ten random hills, which selected from each treatment were counted, and then converted to number of panicles/plant.
5. Number of tillers per plant (NTP): the number of tillers from ten random plants was recorded from the tillers that appeared and grew for each hill.
6. Panicle length (PL cm): it was measured from the collar to the top of the panicle in a sample of ten random panicles.
7. Hundred-grain weight (HGW g): random of 100-rough rice grains from each plot were weighed in grams.
8. Sterility percentage (SET%): the unfilled grains of the main panicle were separated and counted, and sterility percentage was calculated as follows:
  $${\text{Sterility}}\;\% = \frac{{{\text{Number of unfilled grains}}/{\text{panicles}}}}{{\text{Number of total spikelets per panicle}}} \times {1}00$$

### The Drought Tolerance Indices and Genotypes Ranking

All the drought tolerance indices and the ranking of the most drought tolerance genotypes were performed using iPASTIC: an online toolkit to calculate plant abiotic stress indices (Pour-Aboughadareh et al. 2019). The selection of most drought tolerant genotypes was done based on sorted the values of the average sum of ranks (ASR) for each trait. The Venn diagram was created using an integrative tool for comparing lists with Venn diagrams which is online available at http://www.bioinformatics.com.cn/static/others/jvenn_en/index.html.

### Statistical Analysis

Analysis of variance (ANOVA) was performed for all traits using PLABSTAT software (Utz 1997). The following model was used.

$$Y_{ijk} = \mu + y_{i} + t_{n} + r_{j} + g_{k} + gy_{ik} + gt_{ln} + ytrg_{injk} \left( {error} \right)$$

where *Y*_*ij*_ is an observation of genotype *k* in year i and replication *j*, *μ* is the general mean. *t*_*n,*_* y*_*i*_*, r*_*j*_*,* and *g*_*k*_ are the main effects of stress, year, replication, and genotypes, respectively. The error is year × stress × genotype × year interaction of genotype *k* in treatment *t* with year *i*. Replications and years were considered random effects, respectively. Years, replication, and genotypes were considered random effects, while stress was considered as fixed effects. Broad-sense heritability (H) within trials was estimated using HERTI command in PLABSTAT software (Utz 1997). Approximate broad sense heritability (H) across environments was estimated as follows:

$${\text{H}} = \frac{{{\text{VG}}}}{{{\text{VP}}}}$$

Whear VG = Genetic variance (variance due to genetic differences and VP = Phenotypic variance (total variance in the trait in the population).

### The Genotypic Data

The first of its kind in rice, the high-density rice array (HDRA) panel captures the majority of the genetic variation in rice using genotypic data from 1554 accessions. This high-density SNP set afforded much higher resolution than what was previously available and can reveal genetic regions of both minor and major effects (McCouch et al. 2016). Single nucleotide polymorphisms from the HDRA dataset for RDP1 were downloaded from ricediversity.org (http://ricediversity.org/proj/germplasm/index.cfm). These SNPs were filtered for MAF, the percentage missing data, and the percentage of heterozygosity across accessions according to (Alqudaha et al. 2019). As a result, a total of 700,000 SNPs were generated after filtration and used for association study.

### Population Structure

The population structure for the RDP1 was previously done using HDRA dataset containing of 700,000 SNPs and performed by (McCouch et al. 2016). The analysis was done by fast STRUCTURE (Raj et al. 2014).

#### Genome-Wide Association Analysis (GWAS)

The GWAS studies were run using the analysis pipeline and HDRA dataset consisting of 700,000 SNPs described by McCouch et al (2016). In the presented study, GWAS for the eight studied traits (NDH, PH, FLA, NPP, NTP, PL, HGW and SET%) was performed using rMVP R package (Yin et al. 2021) following three different models, Mixed Linear Model (MLM), generalized liner model (GLM), and fixed and random model circulating probability unification (FarmCPU). Kinship (Kin), principal coordinate analysis (PCA), and PCA + Kin were independently included in each model under study to determine which model best matches the trait under study. FarmCPU combines the benefits of mixed linear models and stepwise regression (fixed effect models) and uses them iteratively to fix their drawbacks. In a mixed model (MLM), FarmCPU replaces kinship with a set of markers linked to the causal genes to remove the confounding between kinship and the genes underlying an interesting feature. For testing markers one at a time across the genome, the collection of linked markers is fitted as a fixed effect in a fixed effect model. The associated markers are optimized using a maximum likelihood technique in an MLM with variance and covariance structure determined by the associated markers to prevent model overfitting for testing markers(Liu et al. 2016).The significant markers associated with the studied traits were identified using a *p*-value > 10^−4^.

#### Candidate Genes and Gene Annotation for Studied Traits

To further investigate the genetic control of all traits under the studied conditions, gene models harboring the identified significant markers were investigated by checking the base pair position of the markers and the presence of gene models in the same position using the *EnsemblePlants* database https://plants.ensembl.org/Oryza_sativa/Info/Index The functional annotation of the identified gene models was detected using International Rice Genome Sequencing Project (IRGSP) gene models which imported from the Rice Annotation Project (RAP-DB). The RAP-DB generated a unified assembly of the 12 rice pseudomolecules of *Oryza sativa* Japonica Group cv. Nipponbare. Furthermore, the genetic base of these gene models in relation to drought tolerance was investigated using KnetMiner database https://knetminer.com/cereals/.

## Results

### Phenotypic Performance Within Environments

The mean performance and coefficient of variance for all traits obtained under N and WD for the two growing seasons are presented in Table 2. Observable reduction in all traits under water deficit compared to N condition for FLA, PH, NPT, NPP, PL, and HGW. Highest reduction due to water deficit was found FLA with 39.34% in 2021 and 41.30% in 2022, while, HGW had the lowest reduction with 9.1% in 2021 and 8.8% in 2022. Under water deficit condition, the STE% had a very high increase compared to N with a percentage of 62.36% in 2021 and 61.01% in 2022.

**Table 2:** The mean performance and stander error for the number of days to 50% heading (NDH), flag leaf area (FLA), plant height (PH), no. of tillier per plant (NTP), no. of panicles per plant (NPP), panicles length (PL), hundred grain weight (HGW), and sterility percentage (STE%) for 2021 and 2022 growing seasons under normal irrigation (N) and water deficit condition (WD)

Traits	Years	Irrigation	Means ± Stander error
NDH	2021	WD	106.64 ± 0.86
		N	103.15 ± 0.64
	2022	WD	109.00 ± 0.83
		N	105.13 ± 0.065
FLA	2021	WD	18.76 ± 0.050
		N	30.93 ± 0.058
	2022	WD	18.32 ± 0.043
		N	31.21 ± 0.057
PH	2021	WD	103.94 ± 1.22
		N	141.02 ± 1.52
	2022	WD	104.29 ± 1.23
		N	143.39 ± 1.56
NTP	2021	WD	10.78 ± 0.20
		N	17.25 ± 0.22
	2022	WD	11.04 ± 0.20
		N	17.35 ± 0.22
NPP	2021	WD	10.18 ± 0.20
		N	16.67 ± 0.23
	2022	WD	10.52 ± 0.20
		N	16.88 ± 0.22
PL	2021	WD	18.47 ± 0.17
		N	23.60 ± 0.20
	2022	WD	18.73 ± 0.18
		N	23.81 ± 0.20
HGW	2021	WD	2.38 ± 0.02
		N	2.62 ± 0.02
	2022	WD	2.37 ± 0.97
		N	2.60 ± 0.32
STE%	2021	WD	19.77 ± 0.96
		N	7.44 ± 0.32
	2022	WD	20.03 ± 0.97
		N	7.81 ± 0.32

The analysis of variance (ANOVA) for all traits is presented in Table 3. According to the Bartlett test, variance across the eight traits was homogenous for the two years and across the two stress treatments (WD and N). The ANOVA indicated a significant statistical effect (*p* < 0.01) for the Years (Y), Stress (WD and N), and Genotypes (G) across all traits. Moreover, the combined ANOVA indicated a significant effect for the two- and three-way interactions across all traits except NTP, NPP and PL.

**Table 3:** The analysis of variance for the number of days to 50% heading (NDH), flag leaf area (FLA), plant height (PH), no. of tiller per plant (NTP), no. of panicles per plant (NPP), panicles length (PL), hundred grain weight (HGW) and sterility percentage (SET%) in 2021 and 2022 under N irrigation and WD condition

Source	df	Mean of square							
		NDH	FLA	PH	NTP	NPP	PL	HGW	STE%
Years	1	5543.21**	7.34**	2190.41**	37.60**	89.39**	65.38**	153,196.45**	617,021.62**
Treatments	1	15,918.98**	184,554.02**	1,706,033.41**	48,095.08**	48,579.49**	30,647.47**	42,221.58**	91,904.38**
Replicates	2	14.29*	5.90*	42.09**	91.23**	51.76**	210.59**	12.22**	140.40**
Genotypes	391	1878.00**	652.11**	6770.70**	151.11**	151.03**	87.37**	348.35**	491.02**
Genotypes × Years	391	33.29**	82.96**	5.76**	0.03 ns	0.07 ns	0.01 ns	361.64**	953.73**
treatment x Years	1	41.96**	150.56**	1196.06**	6.77**	5.46**	0.40 ns	45,575.07**	526,871.42**
Genotypes x treatment	391	294.14**	269.96**	884.57**	28.74**	29.34**	53.82**	156.69**	342.31**
Genotypes x treatment x Years	391	32.53**	80.75**	6.54**	0.03 ns	0.06 ns	0.01 ns	156.63**	286.58**
Error	3143	3.31	0.38	4.67	0.40	0.32	0.26	0.25	0.48
Heritability (H^2^)		93.99	81.85	95.77	94.02	93.89	82.94	90.14	87.17

**Significant at *p*-value < 0.05

### Phenotypic Correlation Among all Traits Under WD Conditions

The phenotypic correlation among all traits scored under WD in both growing seasons is presented in Table 4. In the first growing season, a positive significant correlation was found between NDH and PH, FLA, PL, NTP and NPP. The highest correlation values were found between NPP and NTP with r = 0.99** followed by PL and PH with r = 0.34**. While a negative significant correlation was found between HGW and NDH, NTP, NPP, PL and SET. The highest negative correlation was found between HGW and NTP with r = − 0.28**. In the second growing season, the strong positive correlation was found between NPP and NTP with r = 0.99** followed by PH and PL with r = 0.34**. In both years and under WD, significant positive correlations were found between PH and NDH, PH and FLA, PH and PL, NPP and NTP, and NTP and SET. Negative and significant correlations, on the other hand, were found between PH and SET, NPP and PL, NTP and HGW, NPP and HGW, PL and HGW, PL and SET, and HGW and SET under WD stress in both growing seasons. The highest significant correlation was found between NPP and NTP with r = 0.99** in both years.

**Table 4:** Phenotypic correlation among studied traits in 2021 (bold font), among yield traits in 2022 (normal font), and the diagonal values refer to the correlation between each trait under WD condition in the two growing seasons

	NDH	FLA	PH	NTP	NPP	PL	HGW	SET
NDH	0.91**	0.23**	0.30**	0.12*	0.11*	**0.04**	− 0.15**	**0.09**
FLA	0.06	0.25**	0.31**	** − 0.09**	** − 0.09**	0.18**	** − 0.04**	** − 0.09**
PH	0.28**	0.14**	0.99**	** − 0.001**	** − 0.004**	0.34**	**0.03**	− 0.17**
NTP	0.08	** − **0.02	** − **0.004	0.99**	0.99**	− 0.17**	− 0.28**	0.21**
NPP	0.07	** − **0.02	** − **0.007	0.99**	0.99**	− 0.17**	− 0.27**	0.23**
PL	0.04	0.06 ns	0.34**	** − **0.16**	** − **0.17**	0.99**	0.11*	− 0.24**
HGW	** − **0.02	** − **0.04	0.02	** − **0.23**	** − **0.22**	0.02	0.60**	− 0.23**
SET	0.08	** − **0.02	** − **0.17**	0.22**	0.23**	** − **0.24**	** − **0.12*	0.99**

*p value < 0.05, **p value < 0.01 and ns Nonsignificant *p* value

The diagonal values refer to the correlation between each trait under WD condition in the two growing seasons. A strong positive correlation was observed between each trait with its counterparts in the second growing season. The highest correlation values r = 0.99** was shown in the SET%, PH, NPP, and PL in both years while the lowest value r = 0.25** was observed in FLA in the two growing seasons.

### Selection for the Most Promising Drought TOLERANCE Genotypes for the Upcoming Breeding Program

**Fig. 1:**
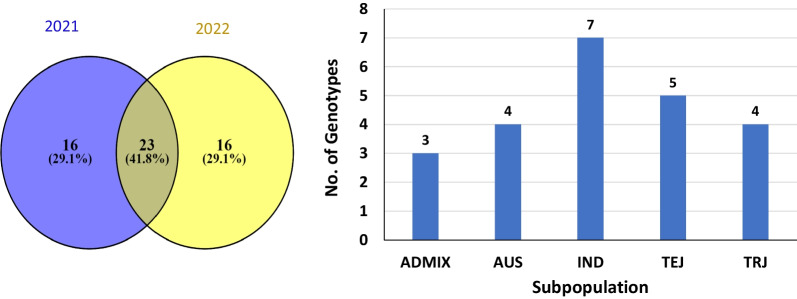
The most drought tolerant genotypes in the two growing seasons (**a**); the distribution of the 23 common genotypes among the five subpopulations (**b**)

Different stress tolerance indices were calculated for all traits to select the most drought tolerant genotypes in both years. The results of each stress selection index for each trait are presented in Additional file 1: Table S2. In each stress index, all genotypes were sorted based on average sum of ranks (ASR), then the highest drought tolerant genotypes were selected. In each growing season, the genotype was finally selected if it was among the best 50 drought tolerant genotype in at least three stress indices. As a result, a set of 39 genotypes were considered drought tolerant genotypes in the two growing seasons. Interestingly, 23 genotypes were common and stable in the two-growing season (Fig. 1a, Table 5). We focused on the 23 genotypes in the following sections. The selected genotypes were from different countries representing West Europe, East Asia, Central America, Southeast Asia, Africa, and South America.

**Table 5:** The individual ID, name, Original providing country, region and subpopulation group according to (McCouch et al. 2016)

GSOR ID†	NSFTV ID††	Name	Original providing country	Region	#Subpopulation group (fastStructure) (McCouch et al. 2016)
301,001	NSFTV_1	Agostano	Italy	West Europe	TEJ
301,012	NSFTV_13	NSF-TV 13	Unknown	Unknown	AUS
301,383	NSFTV_15	Beonjo	Republic of Korea	East Asia	TEJ
301,028	NSFTV_30	Chiem Chanh	Vietnam	Southeast Asia	IND
301,033	NSFTV_35	CO18	India	South Asia	IND
301,057	NSFTV_61	Guan-Yin-Tsan	China	East Asia	IND
301,058	NSFTV_65	Honduras	Honduras	Central America	TRJ
301,061	NSFTV_68	I-Geo-Tze	Taiwan	East Asia	ADMIX
301,072	NSFTV_79	Jouiku 393G	Japan	East Asia	TEJ
301,079	NSFTV_87	Keriting Tingii	Indonesia	Southeast Asia	ADMIX
301,084	NSFTV_92	Kinastano	Philippines	Southeast Asia	TRJ
301,094	NSFTV_102	Leung Pratew	Thailand	Southeast Asia	IND
301,125	NSFTV_134	Romeo	Italy	West Europe	TEJ
301,137	NSFTV_146	Shuang-Chiang	Taiwan	East Asia	IND
301,139	NSFTV_148	Sintane Diofor	Burkina Faso	Africa	IND
301,154	NSFTV_163	Taducan	Philippines	Southeast Asia	IND
301,204	NSFTV_213	WC 3397	Jamaica	Caribbean	TRJ
301,252	NSFTV_262	Halwa Gose Red	Iraq	West Asia	AUS
301,269	NSFTV_279	Kon Suito	Mongolia	East Asia	TEJ
301,347	NSFTV_359	Surjamkuhi	India	South Asia	AUS
301,348	NSFTV_360	PTB 30	India	South Asia	AUS
301,361	NSFTV_376	Breviaristata	Portugal	West Europe	ADMIX
301,364	NSFTV_379	Wanica	Suriname	South America	TRJ

^†^GSOR: Genetic Stocks-Oryza collection identification number

^††^NSFTV: National Science Foundation-"Exploring the Genetic Basis of Transgressive Variation in Rice" project accession identification number

^#^Subpopulation identified by fastStructure analysis based on 700,000 SNPs [McCouch et al.; Nature Communications (2016)7:10532]

Aus is coded with AUS, indica with IND, temperate japonica with TEJ, tropical japonica with TRJ and admixed with ADMIX

The population structure which previously done by (McCouch et al. 2016) using fast STRUCTURE were divided the RDP1 into five subpopulation. The five subpopulations were named aus, indica, tropical, temperate, and admixed (ADMIX) japonicas. Figure 1b shows the distribution of the 23 common genotypes among the five subpopulations. The Indica subpopulation has the highest number of common genotypes (7), followed by the temperate japonica TEJ subpopulation with 5 genotypes, the tropical japonica TRJ and AUS subpopulation with 4, and the ADMIX subpopulation with 3 genotypes.

### Genome Wide Association Study

**Fig. 2:**
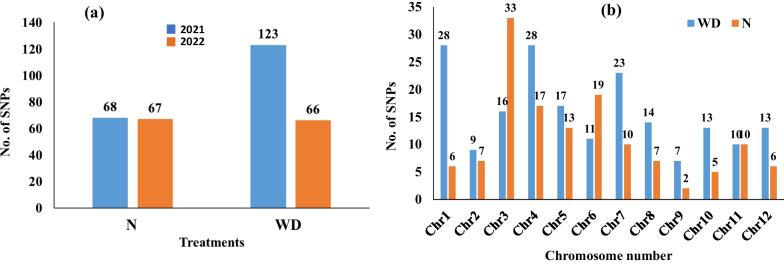
The number of SNP markers associated with each trait in the two water treatments in 2021 and 2022 (**a**); The distribution of 324 significant markers across all the rice chromosomes (**b**)

The analysis of GWAS revealed 340 significant SNPs associated with all traits in both growing seasons under both conditions. In both conditions, the QQ-plot results represented that the best GWAS models for all the traits was FarmCPU the Q-Q plots were presented in Additional file 1: Fig. S1and S2. An approximate number of significant SNPs were found under N in both years, while the number of significant SNPs were higher in 2021 (123 SNPs) than those detected in 2022 (23 SNPs) under WD (Fig. 2a). The distribution of all 324 significant SNPs across all the rice chromosomes was presented in (Fig. 2b). Under N conditions, the highest number of significant SNPs was observed on chromosome 3 (33 SNPs), while, chromosomes 1 and 4 had the highest number of significant SNPs (28 SNPs) under WD. Chromosome 9 had the lowest number of significant SNPs under both conditions.

**Fig. 3:**
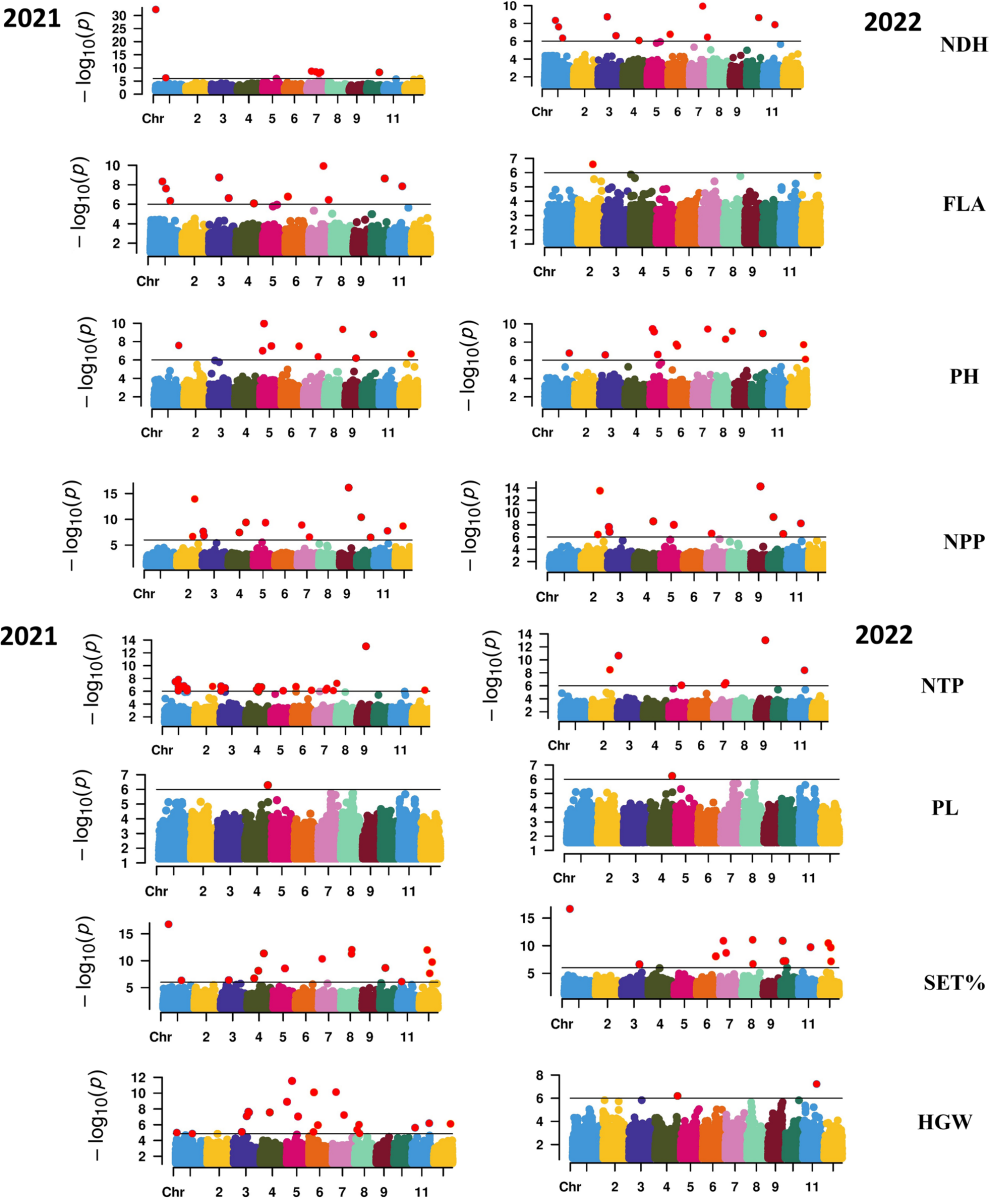
Manhattan plot for all traits scored under drought stress in the two growing seasons of 2021 and 2022

In 2021, the GWAS found a total of 191 significant SNPs under both conditions Additional file 1: Table S3 and the summarize GWAS results are presented in Table 6. The manhattan plot for all traits scored under WD conditions in the two-growing season of 2021 and 2022 were presented in Fig. 3. The Manhattan plot for all traits scored under N conditions in the two-growing season of 2021 and 2022 were presented in Additional file l: Fig. S3. NTP had the highest number of significant SNPs (42 SNPs), while one SNP was found to be significant associated with PL under WD. A set of 12 significant markers were detected for FLA under N, while only five SNP markers were detected for PL. One shared marker SNP-12.24590895 (chr. 12) was detected under both conditions. The allele T of this SNP marker was found to be associated with increased HGW under both conditions.

**Table 6:** The significant SNPs associated with NDH, FLA, PH, NTP, NPP, PL, HGW and SET% under WD and N irrigation in both growing season

Stress	Trait	No. of SNPs	− log10 *P* value		Allele effect	
			Min.	Max.	Min.	Max.
*Season 2021*						
WD	NDH	9	5.15 × 10^−33^	1.10 × 10^−6^	− 106.50	5.85
N		11	9.54 × 10^–20^	5.50 × 10^–7^	− 3.82	8.24
WD	FLA	19	2.10 × 10^–8^	1.19 × 10^–6^	7.91	27.88
N		12	1.37 × 10^–7^	1.18 × 10^–6^	− 7.17	4.64
WD	PH	11	1.07 × 10^–10^	1.14 × 10^–6^	− 9.77	11.56
N		7	8.76 × 10^–11^	1.37 × 10^–6^	− 9.33	35.80
WD	NTP	42	1.44 × 10^–8^	1.41 × 10^–6^	− 2.06	8.61
N		8	3.50 × 10^–8^	1.12 × 10^–6^	1.74	4.33
WD	NPP	11	7.35 × 10^–17^		2.69 × 10^–7^	
N		8	4.39 × 10^–8^	1.14 × 10^–6^	1.74	4.33
WD	PL	1	5.14 × 10^–7^		− 1.97	
N		5	2.96 × 10^–7^	1.17 × 10^–6^	1.64	4.10
WD	HGW	14	2.83 × 10^–12^	1.12 × 10^–6^	− 0.86	0.91
N		7	1.14 × 10^–10^	7.34 × 10^–7^	− 0.16	0.26
WD	SET%	16	1.70 × 10^–17^	1.40 × 10^–6^	− 6.31	47.02
N		10	2.30 × 10^–24^	9.31 × 10^–7^	− 1.01	11.27
*Season 2022*						
WD		12	1.18 × 10^–10^	1.13 × 10^–6^	− 17.46	26.03
N	NDH	8	2.24 × 10^–15^	4.58 × 10^–7^	3.39	26.48
WD	FLA	2	2.6 × 10^–7^	1.35 × 10^–6^	3.10	6.62
N		11	1.36 × 10^–7^	1.37 × 10^–6^	− 6.19	4.56
WD	PH	13	3.54 × 10^–10^	7.65 × 10^–7^	− 20.02	11.83
N		7	1.46 × 10^–11^	6.98 × 10^–7^	− 17.81	10.47
WD	NTP	7	9.49 × 10^–14^	8.44 × 10^–7^	− 0.94	7.03
N		8	3.57 × 10^–8^	1.12 × 10^–6^	1.74	4.26
WD	NPP	10	6.16 × 10–^16^	1.14 × 10^–6^	− 1.16	7.38
N		7	2.71 × 10^–8^	1.07 × 10^–6^	1.73	4.17
WD	PL	1	5.84 × 10^–7^		− 1.97	
N		5	3.06 × 10^–7^	1.28 × 10^–6^	1.65	4.13
WD	HGW	5	5.95 × 10^–8^	1.16 × 10^–6^	0.159	0.308
N		6	1.25 × 10^–7^	1.14 × 10^–6^	0.123	0.18
WD	SET%	16	2.19 × 10^–17^	1.17 × 10^–6^	− 2.59	35.31
N		15	5.60 × 10^–20^	8.04 × 10^–7^	− 1.28	9.92

A total of 133 SNP markers were found to be associated with yield traits in 2022 under both conditions Additional file 1: Table S4 and the summarize GWAS results are presented in Table 6. The manhattan plot for all traits scored under N conditions in the two-growing season of 2021 and 2022 were presented in Additional file 1: Fig. S1. SET% had the highest number of significant SNPs (16) under WD, while one marker was detected for PL. Under N, on the other hand, five significant SNPs were found to be associated with PL in 2022. One shared marker (SNP-11.20585882.) located on chr 11. The allele T of this SNP was significantly associated with increased HGW with approximately the same effect (0.18 g) under both conditions.

**Fig. 4:**
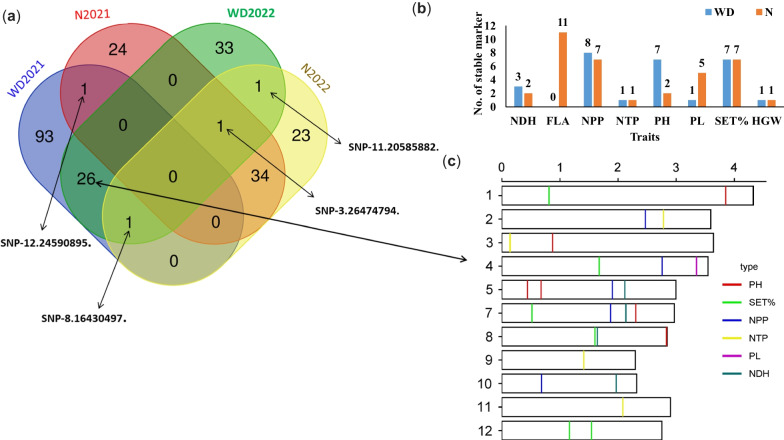
The Venn diagram represents the number of common markers in both growing seasons under Normal (N) and water deficit (WD) conditions (**a**); The number of significant validated and stable markers in the both growing seasons 2021 and 2022 under N and WD conditions (**b**). The distribution of 26 common makers across all the rice chromosomes except Chr. 6 (**c**)

By considering the four conditions, one SNP marker (SNP-3.2647479) was found to be associated with N2022, N/2022, and WD/2022. Moreover, SNP-8.16430497 was found to be associated with SET% under WD/2021 (SET%) and WD/2022(SET%), NDH (N/2022) (Fig. 4a). The number of significant validated and stable markers in both growing seasons 2021 and 2022 under WD and N conditions are presented in Fig. 4b. Under N conditions the FLA showed the highest number of validated and stable markers (11 SNPs) in both growing seasons. On the other hand, no markers are validated in the FLA under WD conditions. In the SET% 7 validated and stable markers were reported in both growing seasons under WD and N conditions. The lowest number of validated markers (1 SNP).

**Fig. 5:**
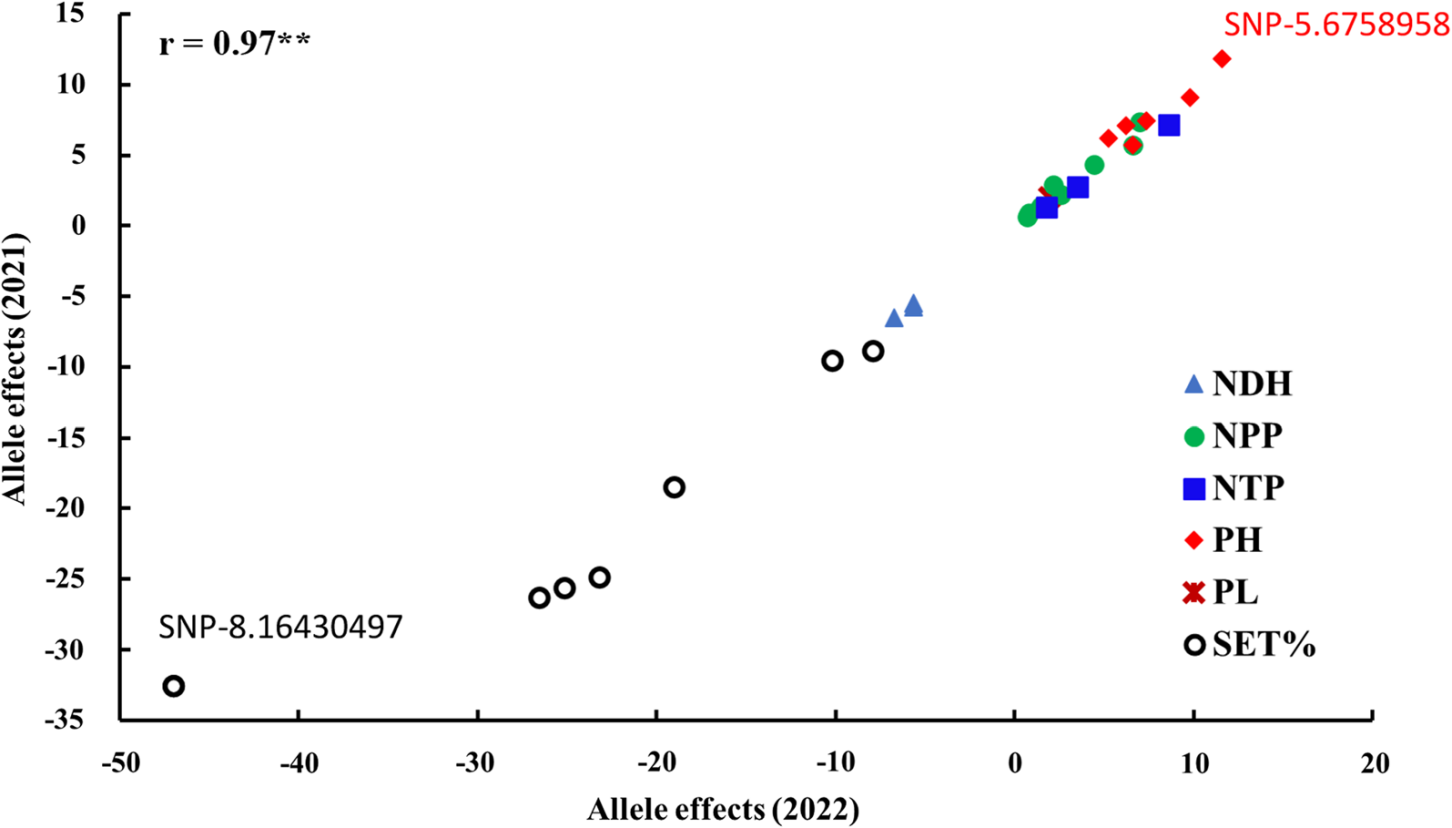
The allele effect of the 26 common markers in both growing seasons under water deficit conditions (WD)

In this study, we focused on the 26 common markers detected under WD in both growing seasons in the next sections. The list of shared significant markers between the two years under stress is presented in Additional file 1: Table S5. The 26 common markers were distributed on all chromosomes except Chr. 6 Fig. 4c. Chromosome 8 had the highest number of common significant markers, while two common SNPs were found to be located on chromosome 1, 9, 10, 11, and 12. The linkage disequilibrium among markers located on the same chromosome was calculated. No significant LD was found among SNP pairs located on the same chromosome (data not shown). Interestingly, each common marker was found be associated with the same trait except three markers five markers that were associated with the same trait in both years in addition to a third different trait. For example, SNP-8.16430497 marker was significantly associated with NDH in both years and with SET% under WD/2022. NPP had the highest number of common markers (9), while PL had only one common makers in both growing seasons. The allele effect of the 26 common markers in both growing seasons under WD was investigated to see the stability of these markers on the traits (Fig. 5). High significant correlation was found between the allele effects between 2021 and 2022 under WD with r = 0.97**.

## Discussion

### Genetic Variation in Yield Traits Under Normal and Water Deficit Conditions

The productivity of most field crops is significantly impacted by abiotic conditions like drought. Depending on the timing, duration, and severity of the drought, drought stress can occur at any stage of a crop's growth and have varying effects on productivity (Mondal et al. 2021; Mourad et al. 2019; Sallam et al. 2019). It has been noted that drought stress directly lowers production during the reproductive stage of plants (Table 7), as well as typically suppressing plant growth during the vegetable stage (Ma et al. 2016; Yue et al. 2006). Although agronomists and breeders have made significant progress towards making crops more drought tolerant, the genetic and molecular bases of drought resistance in crops are still largely unclear (Ahmed et al. 2021; Sallam et al. 2022).

**Table 7:** The gene ID, gene name coding protein and the biological process for the 16 SNPs

Year	Stress	Trait	SNP ID	Gene ID	Gene Name	Protein coding	Biological process
2021	WD	NPP/NTP	SNP-3.1410415	Os03g0124300	FER	Receptor-like protein kinase FERONIA-like	Response To Water Deprivation
2022							
2021	WD	PH	SNP-3.8751808	Os03g0265300	PXN	Peroxisomal Transport	Primary and secondary metabolism, development, and responses to abiotic and biotic stresses
2022							
2021	WD	SET%	SNP-4.16583620	Os04g0348300	CDC5	Pre-mRNA splicing factor component CDC5L/Cef1	Regulation Of Protein Localization
2022							
2021	WD	PL	SNP-4.33316160	Os04g0653200	OSCAX3	Calcium/proton exchanger	Ion Transport
2022							
2021	WD	NDH	SNP-10.19624308	Os10g0510400	AdoMet	Putative S-adenosyl-L-methionine-dependent methyltransferase	Response To Water Deprivation
2022							
2021	WD	NPP/NTP	SNP-11.20345396	Os11g0549620	PAP3	Purple acid phosphatase	Response To Water Deprivation
2022							
2021	N	SET%	SNP-2.24141959	Os02g0612800	BIP135	Sister chromatid cohesion protein Pds5	Mitotic Sister Chromatid Cohesion
2022							
2021	N	PL	SNP-3.15006885	Os03g0379500	RPS9	Ribosomal protein S4/S9	Positive Regulation of Translational Fidelity
2022							
2021	N	FLA	SNP-3.27988841	Os03g0698300	MTs	Transmembrane Transporter Activity	Water and nutrient movement and improving tolerance mechanisms against numerous abiotic stresses Regulation Of Developmental Growth
2022							
2021	N	FLA	SNP-3.27992294	Os03g0698350	ADCK	ADCK1-like domain	
2022							
2021	N	FLA	SNP-3.28018218	Os03g0698900	ACER	Alkaline ceramidase, plant	Important functions in plant growth and stress responses
2022							
2021	N	FLA	SNP-3.28135195	Os03g0701000	emb2734	Importin beta family	Nucleocytoplasmic transport receptors
2022							
2021	N	FLA	SNP-3.28220322	Os03g0702500	UGT91D2	UDP-glucuronosyl/UDP-glucosyltransferase	Role in conferring drought tolerance
2022							
2021	N	PH	SNP-6.3158894	Os06g0162700	MYB98	SANT/Myb domain	Regulation of a plant-specific developmental program
2022							
2021	N	NPP/NTP	SNP-6.422802	Os06g0106500	PLCP	Papain-like cysteine peptidase superfamily	Protein proteolysis and involved in numerous physiological processes
2022							
2021	N	SET%	SNP-6.682168	Os06g0112100	BSPA	Catalytic Activity	Nucleoside Metabolic Process
2022							

In this study, highly diverse rice population showed high genetic variation in yield-related traits under water deficit. Such genetic variation is very useful for rice breeders to select promising drought tolerant rice genotypes as candidate parents for breeding program. Screening a large plant germplasm for target traits is very important to precisely improve crop production through breeding program (Amro et al. 2022; Bhandari et al. 2021; Abou-Zeid and Mourad 2021). The ANOVA revealed highly significant G x E interaction indicated that genotypes performed differently in both years under N and WD conditions. Therefore, selection based on different stress indices for drought tolerance was performed each year separately. Drought-tolerant genotypes in each single trait for all stress indices were considered and superior genotypes were selected if they had a high performance in at least four stress indices. Selection based on multiple traits is highly performed than single-trait selection to obtain the true promising candidate genotypes for future breeding program (Ghazy et al. 2021; Raman et al. 2012). As a result, a set of 23 drought tolerant rice were selected due to their high yielding attributed in both years. These genotypes represented different countries and different subpopulations (Fig. 1b), indicating that these genotypes also have a high genetic diversity among them. Crossing highly divergent drought-tolerant genotypes is very important to produce cultivars having higher degree of drought tolerance in rice. Ghazy et al. 2021, utilize the analysis of genetic diversity, QTL, and genetic variation in a set of 22 rice cultivars and select the most diverse and high tolerant rice genotypes for future crossing. Therefore, investing genetic diversity in parallel with genetic variation in target trait will help plant breeder to accelerate breeding programs to genetically improve target traits (Eltaher et al. 2018; Mourad et al. 2020; Salem & Sallam 2016).

It was noted the SET% was the most trait affected by WD stress with an increase reached to 61% on average in both years. Drought stress leads to increase spike fertility due to the increase of reactive oxygen species (ROS) levels (Selote & Khanna-Chopra 2004). Highly resistant rice genotypes tend to have a high efficient mechanism that protect them from oxidative conditions (Selote & Khanna-Chopra 2004). Therefore, SET% is an important trait that should be considered to highly determine drought tolerant genotypes.

### Genome-Wide Association Study (GWAS)

In this study, a set of 413 highly diverse rice genotypes were used to detect maker- association through GWAS. It was previously reported that 100–500 individual are required for genome-wide studies (Alqudah et al. 2020). The use of a diverse panel in GWAS not only helps to map relationships between traits and DNA polymorphisms but also enables us to understand the genetic basis of genetic correlations among phenotypic traits, i.e., pleiotropy versus genetically linked genes, and makes it easier to choose donors with a mix of traits that are likely to be adaptive and selectively advantageous for breeding in target environments. The genotypes and SNP markers used in the current study were previously investigated to identify QTLs associated with phenotypic performance under cold stress conditions in the USA (Shakiba et al. 2017). This study has sufficient power, given our marker density and sample size, to identify alleles with big effects that are shared across populations, but a larger panel with a greater density of SNPs might enable us to identify more QTLs with small effects.

The analysis of the population structure for the RDP was previously performed by McCouch et al. (2016). In GWAS studies, population structure should be considered to prevent spurious association. Therefore, PCA, kinship, and PCA + kinship was included with GLM, MLM, and FarmCPU. Bhandari et al. 2020, draw findings that are based on recent research showing that multi-locus methods—particularly Farm-CPU—are more effective than single-locus methods (like MLM) for analyzing associations between traits that have high or low heritability. This is because they effectively control for false positives and negatives, as evidenced by the sharp deviations seen in the *p*-value distribution in qq plots (Kaler & Purcell 2019; Xu et al. 2018). Based on q-q plot results, FarmCPU was the best GWAS model for all traits scored in this study (Lawson et al. 2020).

Notably, some of the strongest signals can be found quite a distance from known candidate genes. The optimal tag-SNP for a candidate gene may be quite far from the expected locus due to ascertainment bias, or we may be tagging previously unknown loci that just so happen to map close to a known candidate (Zhao et al. 2011).

The GWAS of this study revealed very important SNP markers associated with yield traits under both conditions and the two growing seasons. It was observed that the number of QTL detected in 2021 under both conditions was higher than those detected in 2022. This was due to the effect of environment (year) on identify significant SNPs associated with target traits (Eltaher et al. 2021a). Therefore, it is highly recommended to test the same population over locations or years to truly identify significant markers. Recently, 17 QTLs were linked to drought tolerance in the vegetative stage when grown in a greenhouse, according to GWAS research done on 180 Vietnamese rice landraces (Hoang et al. 2019). Using a mixed model approach with structure control and kinship among the landraces under research, different significant MTAs were found in the two subpanels of the study, indica and japonica. For improved adaptability in the dry direct seeded rice (DDSR) system, GWAS conducted by (Subedi et al. 2019) revealed 37 highly significant MTAs for 20 parameters, including plant and root morphological traits, nutrient uptake, yield, and its components in MAGIC population of 5 different parents. Wang et al. )^2023^(, identified 78 SNPs significantly associated with drought tolerance in the 305 accessions, including 15, 33, 17, and 13 SNPs associated with grain number per plant (GYP), grain number per panicle (GNP), panicle number per plant (PNP), and plant height (PH), respectively. All these 78 significant SNPs were tagged to only 42 QTLs distributed on all 12 chromosomes, which agrees with our finding.

A set of shared 34 and 26 significant SNPs were found in both years under N and WD, respectively. These markers can be considered as stable markers; however, they should also be tested in further genetic backgrounds before using them in MAS. Genetic validation testes whether the same marker (or QTL) or candidate gene is likely to be significantly detected when the plant material is evaluated in other years or different locations (Eltaher et al. 2021b; Hashem et al. 2023; Sallam et al. 2016, 2022, 2023). Notably, no significant marker was found in the four environments: N2021, N2022, WD2021, and WD2022. However, four markers were significant under three environments (Fig. 4a). These markers could be specifically tested in different genetic backgrounds under both conditions.

The 26 significant markers detected under WD in both growing seasons provide very important information that can be utilized to genetically improve yield traits under WD conditions. These markers were found to be associated with PH, NPP, SET%, NTP, and NDH. The NDH and SET% had the highest number of shared markers in both years. Out of 26 markers, five were found to be associated with more than one trait, indicating that these markers had pleiotropic effects. Markers with pleiotropic effects are very useful in marker-assisted selection. Of the five markers with pleiotropic effects, four (SNP-11.20345396., SNP-2.27812486, SNP-9.14101710., and SNP-3.1410415 were associated with NTP and NPP. Shared markers between these two traits were expected due to the strong phenotypic correlation between NTP and NPP. Only one marker SNP-8.16430497 was found to be associated with NDH and SET% although no significant correlation between them. Gene annotation of the 26 SNPs was investigated. Only six SNPs were found to be located within six different gene models (Table 7). SNP-3.1410415. was found to be located within LOC_Os03g03290 gene model which encodes Receptor-like protein kinase FERONIA-like. The receptor-like kinase FERONIA was previously found to be tightly involved in plant development, plant growth, responses to various stresses (Jing et al. 2023). In apple plants, the FERONIA receptor kinase was associated with induced abscisic acid under different drought treatments. The activity of receptor-like protein kinase FERONIA-like was associated with less photosystem damage and higher photosynthetic rates under drought conditions (Jing et al. 2023). Also, Receptor-like kinase OsSIK1 was found to significantly improve drought and salt stress tolerance in rice through the activation of the antioxidative system (Ouyang et al. 2010). SNP-3.8751808. markers were located within LOC_Os03g15860 which encodes NAD/FAD transporter SLC25A32-like/Peroxisomal Transport. SNP-4.33316160. was located within LOC_Os04g55940 gene model that encodes calcium/proton exchanger. Ca2+ regulates the physiological response to drought stress by acting as a secondary messenger and transmitting drought signals (Hong-Bo et al. 2008). Moreover, cytosolic-free calcium plays a vital role in the movement of stomata and the regulation of the closing and opening of the stomata (Wang et al. 2005). in Wheat seedlings, it was noted that with the increase in drought duration, the concentration of free Ca2+ in the nucleus was increased, indicating the potential role of the Ca^2+^ in maintaining nucleus structure and integrity (Song et al. 2008). The candidate gene LOC_Os04g28090 (SNP-4.16583620.) that was found for SET% under both years encodes Pre-mRNA splicing factor component CDC5L/Cef1. The LOC_Os10g36690 gene model that included SNP-10.19624308 SNP marker (NDH) encodes putative S-adenosyl-L-methionine-dependent methyltransferase. The protein was highly increased under drought stress in coffee leaves (Mofatto et al. 2016) and maize roots (He et al. 2016). SNP-11.20345396 (NPP and NTP) marker was located within LOC_Os11g34720 gene model which encodes purple acid phosphatase (PAP). In rice, the PAP was found to be associated with pollen development and phosphorus Pi starvation (Deng et al. 2020). It was also reported that the PAP interacts with other genes to alleviate the effect of drought stress on Arabidopsis (.e.g. *AtGAL1*) (Ghahremani et al. 2019) and wild plants (*RAP2*.2)(H. Xu et al. 2022; Zhang et al. 2008). The strong association between the biological function of candidate genes and drought tolerance indicates the successfulness of our GWAS in identifying markers associated with yield traits under drought stress in rice. Also, the candidate genes for SNPs (10 SNPs) associated with the same traits in the both growing season under normal conditions is presented in Table 7.

The LD among SNPs located on the same marker was calculated and non-significant LD was found between any marker pairs, indicating that each significant SNP represented individual QTL. Interestingly, the allele effects of 26 markers were compared in the two growing seasons under WD stress. High significant correlation (r = 0.97**) was found among the allele effects of the 26 marker between 2021 and 2022 indicating the stability of these markers regarding to their effects on the traits. In both growing seasons, the allele T in SNP-5.6758958 was found to be associated with increased PH in 2021 (11.5 cm) and 2022 (11.8). The allele G in SNP-8.16430497 was found to be associated with decreases SET% in 2021 ( − 47.01%) and 2022 (32.54%).

## Conclusion

Large-scale resequencing-based genome-wide association studies (GWAS) offer an efficient means for finding genetic variants that can be utilized to improve crop quality, including drought tolerance. High resolution GWAS has been effectively applied to identify relationships involving complex variables from different collections of rice cultivars with genetic variants. GWAS offers high-resolution genetic mapping that can filter the related regions to potential genes by using high density SNPs at the whole-genome level. This result further supports the usefulness of utilizing such markers to improve drought tolerance in rice. These markers had many advantages (1) they were able to be significantly detected in both growing season under WD stress, (2) each marker was found be associated with the same train, and (3) the effect of target allele of these markers was stable over the two years. Therefore, these markers can be converted to Kompetitive allele specific PCR (KASP) markers to be validated in a different genetic background tested for yield traits under WD stress.

### Supplementary Information

**Additional file 1: Supplementary Figure 1**. QQ plots for all traits scored under normal condition in the two growing season of 2021 and 2022.** Supplementary Figure 2**. QQ plots for all traits scored under water deficit condition in the two growing season of 2021 and 2022.** Supplementary Figure 3**. The Manhattan plot for all traits scored under FI conditions in the two-growing season of 2020 and 2021.** Supplementary Table 1**. List of 392 O. sativa accessions and country of origin, principle component structure and fast structure assignment. **Supplementary Table 2.** the average sum of ranks for all 392 genotypes in each trait at the two growing seasons.** Supplementary Table 3.** the significant SNPs associated with NDH, FLA, PH, NTP, NPP, PL, HGW and SET% under DI and FI irrigation at the first grwoing season (2021).** Supplementary Table 4.** the significant SNPs associated with NDH, FLA, PH, NTP, NPP, PL, HGW and SET% under DI and FI irrigation at the second grwoing season (2022).

## Data Availability

The datasets presented in this study can be found in Additional file 1.
