# Supplementary material for: Genome-Wide Association Study of Rice Diversity Panel Reveals New QTLs for Tolerance to Water Deficit Under the Egyptian Conditions
Source: Rice (N Y). 2024 Apr 23;17:29. doi: 10.1186/s12284-024-00703-1 (PMC11035518; doi:10.1186/s12284-024-00703-1)
Supplement: Supplementary file 1 — Additional file 1: Supplementary Figure 1. QQ plots for all traits scored under normal condition in the two growing season of 2021 and 2022. Supplementary Figure 2. QQ plots for all traits scored under water deficit condition in the two growing season of 2021 and 2022. Supplementary Figure 3. The Manhattan plot for all traits scored under FI conditions in the two-growing season of 2020 and 2021. Supplementary Table 1. List of 392 O. sativa accessions and country of origin, principle component structure and fast structure assignment. Supplementary Table 2. the average sum of ranks for all 392 genotypes in each trait at the two growing seasons. Supplementary Table 3. the significant SNPs associated with NDH, FLA, PH, NTP, NPP, PL, HGW and SET% under DI and FI irrigation at the first grwoing season (2021). Supplementary Table 4. the significant SNPs associated with NDH, FLA, PH, NTP, NPP, PL, HGW and SET% under DI and FI irrigation at the second grwoing season (2022). [file 12284_2024_703_MOESM1_ESM.zip › Supplementary Figure 2.pdf]

FLA

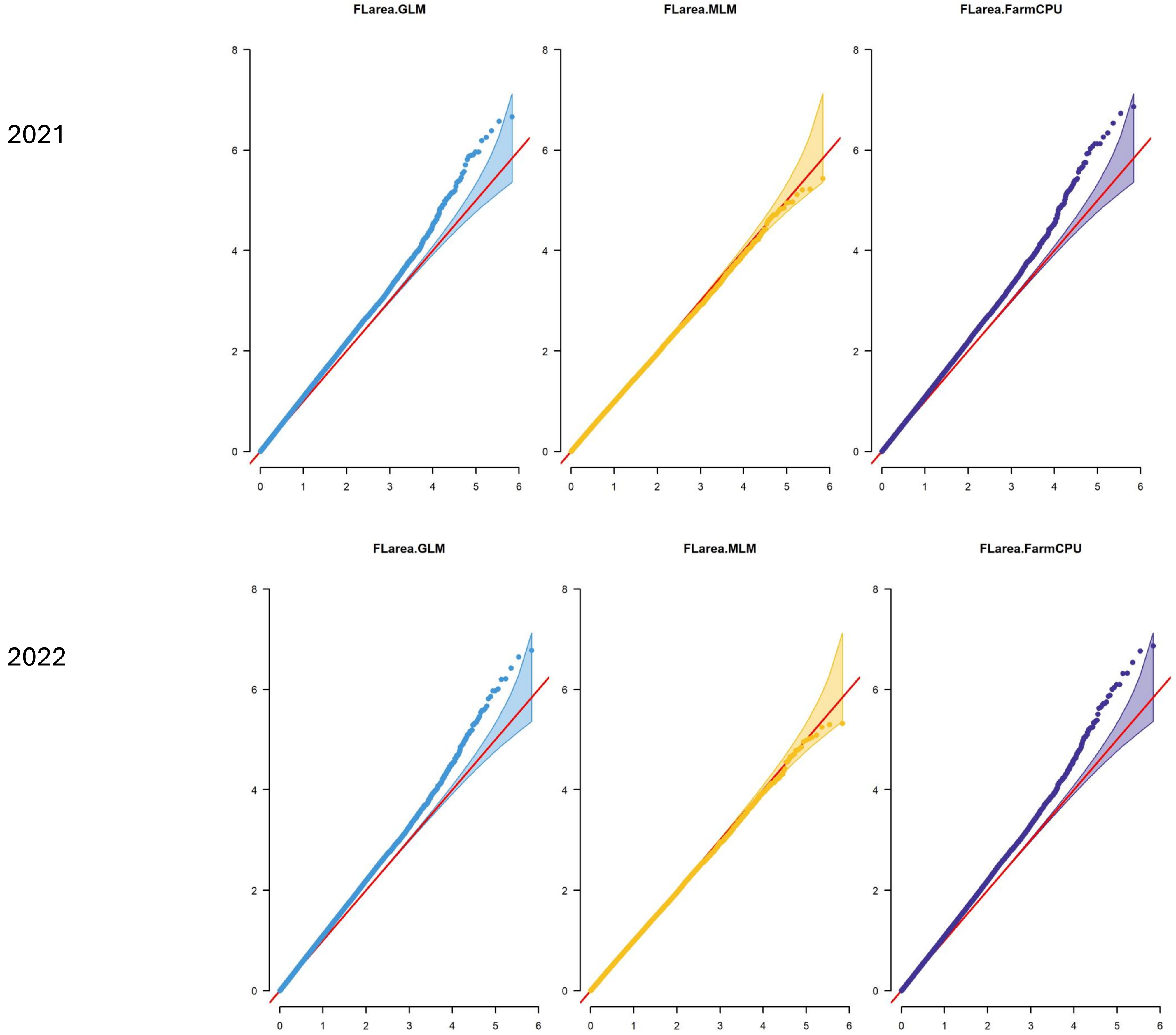
2021
FLarea.GLM
FLarea.MLM
FLarea.FarmCPU
2022
FLarea.GLM
FLarea.MLM
FLarea.FarmCPU

NDH

2021

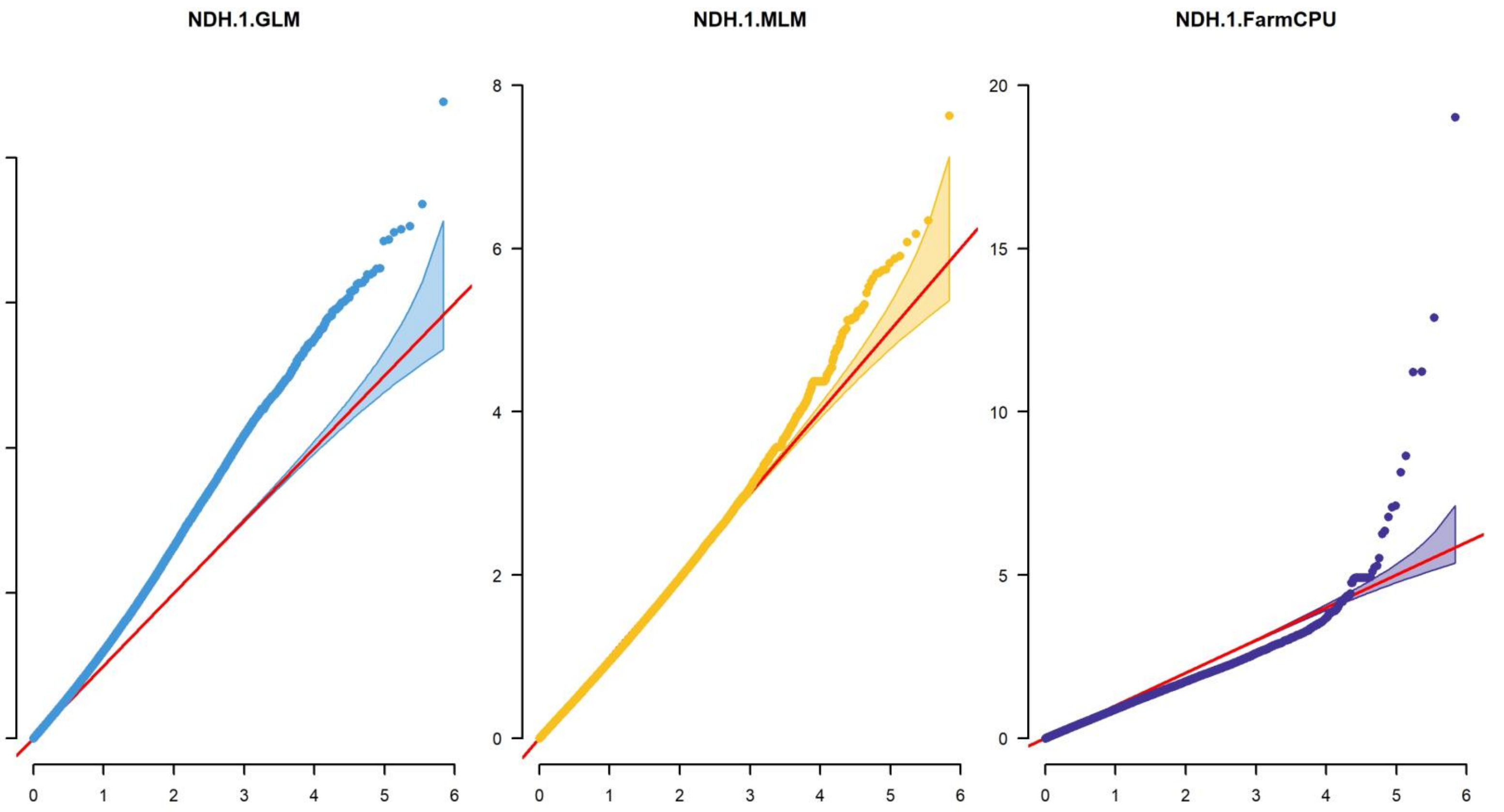

2022

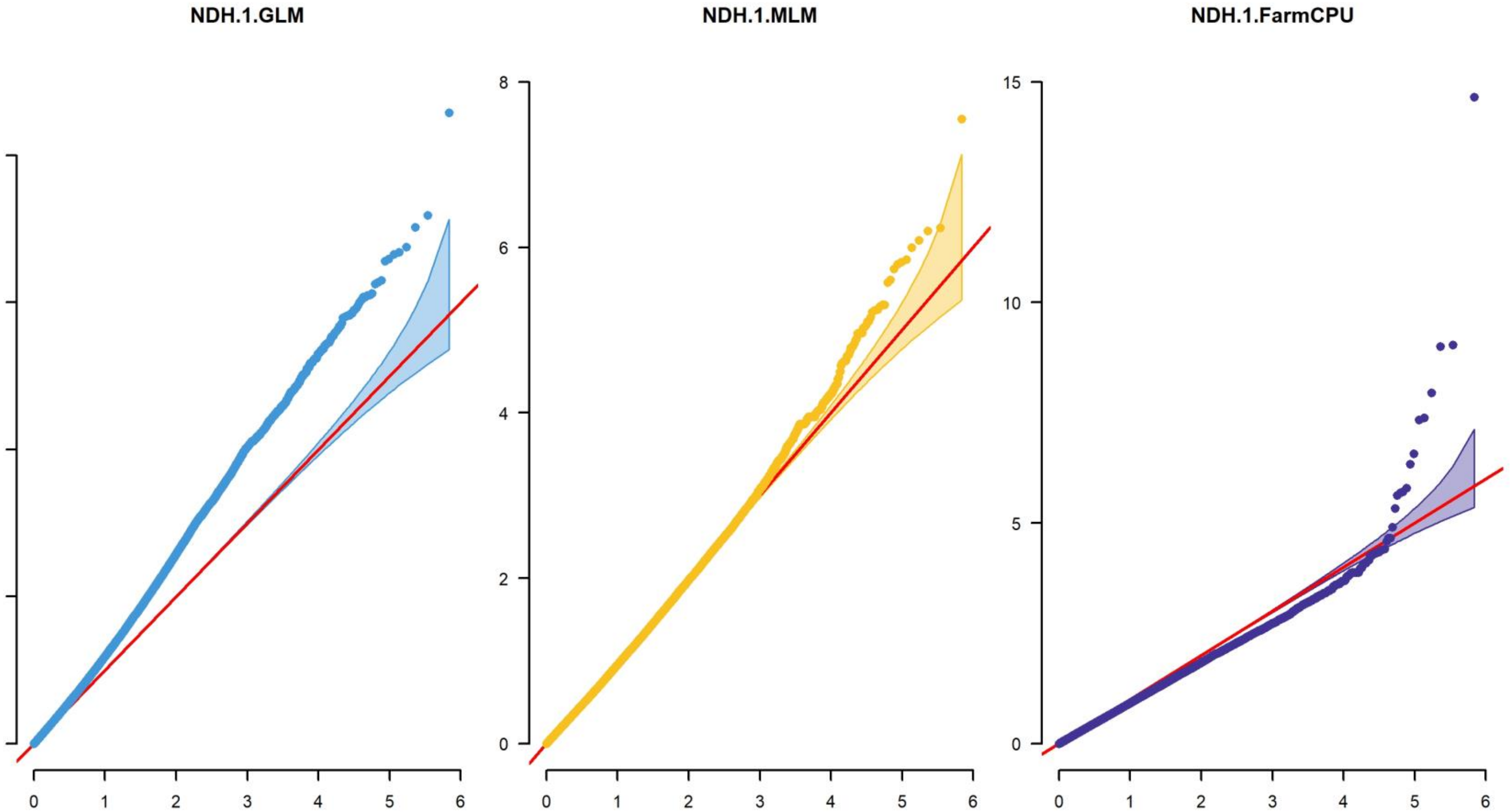

NPP

2021

2022

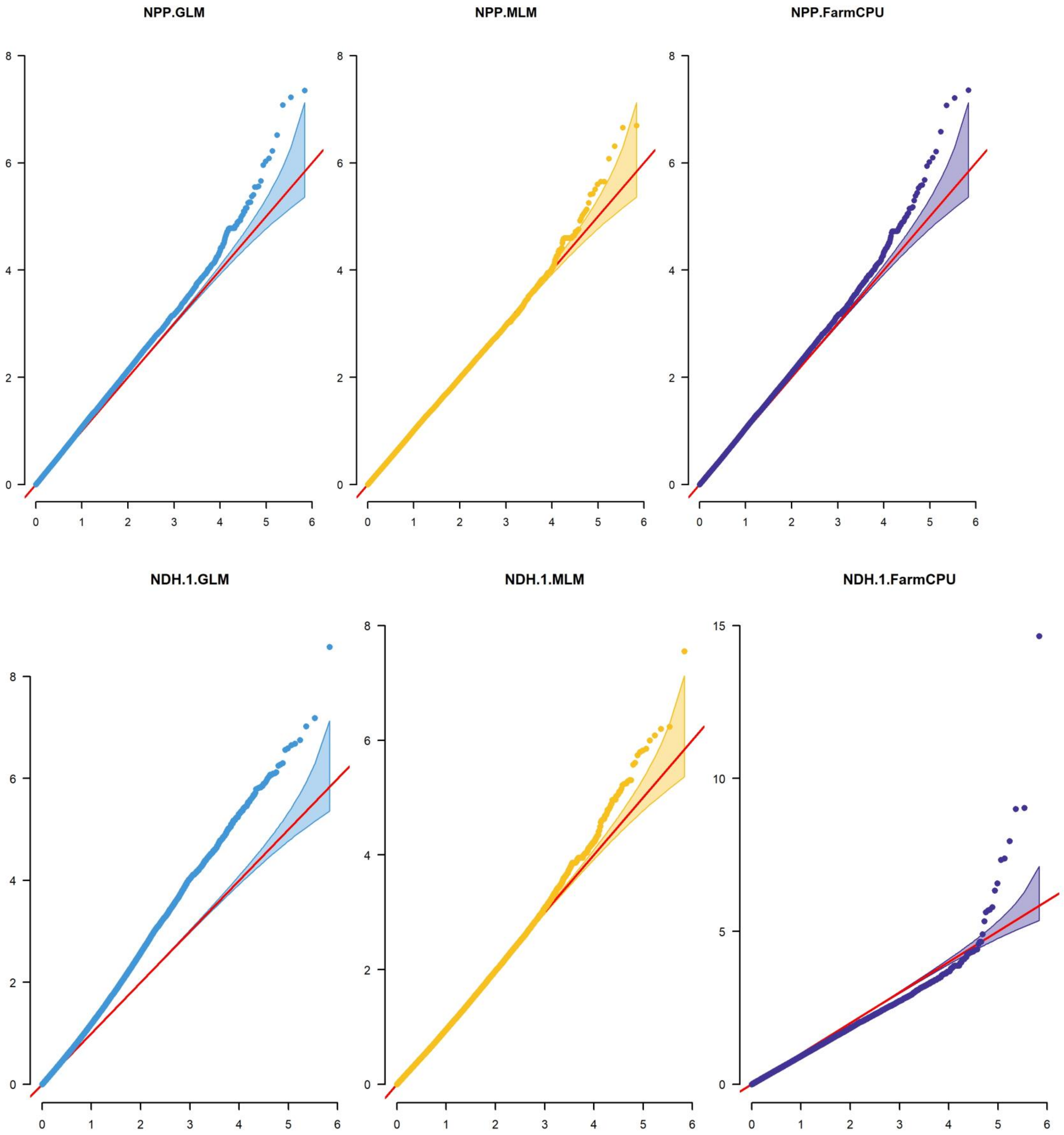

NTP

2021

2022

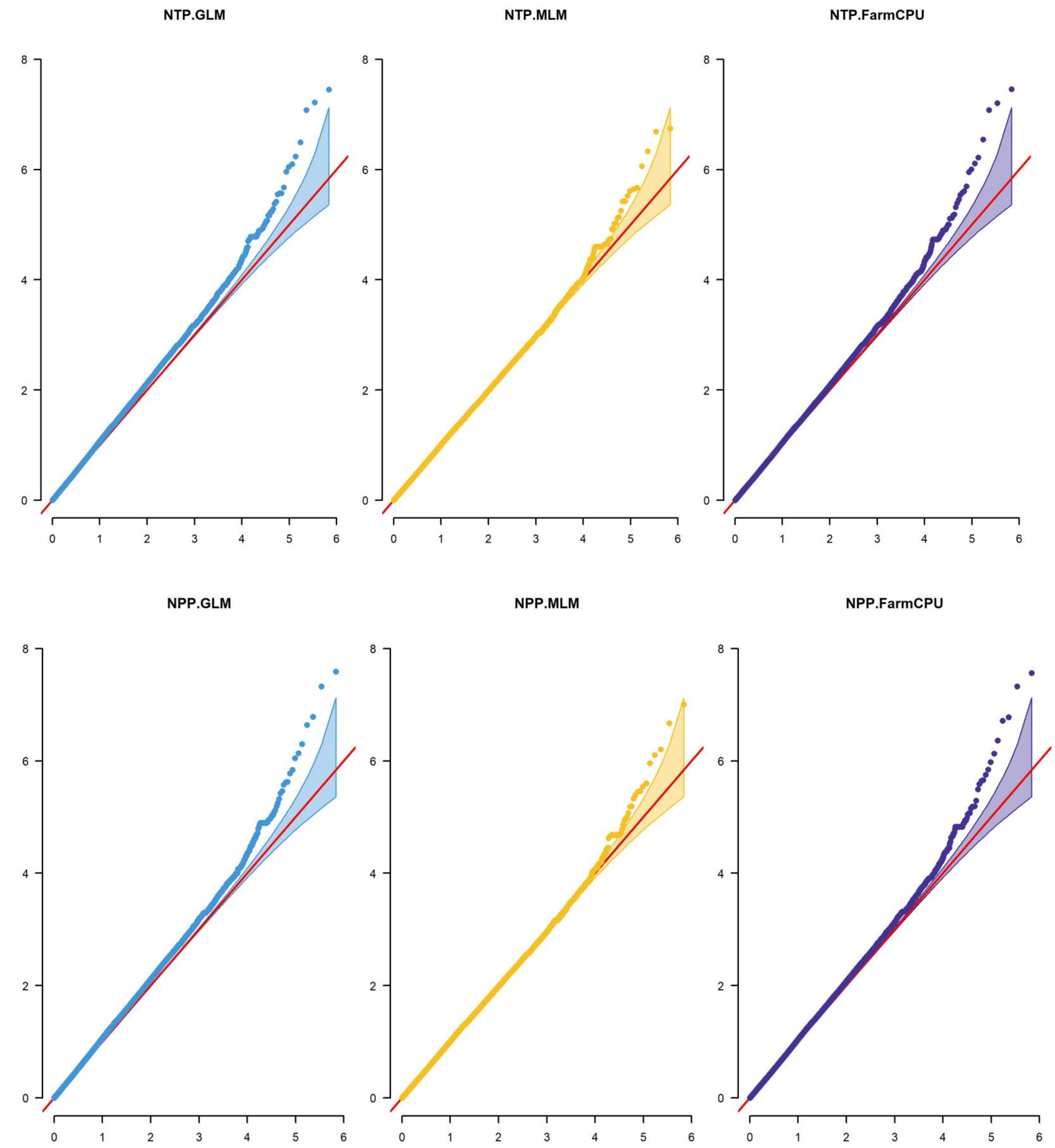

PH

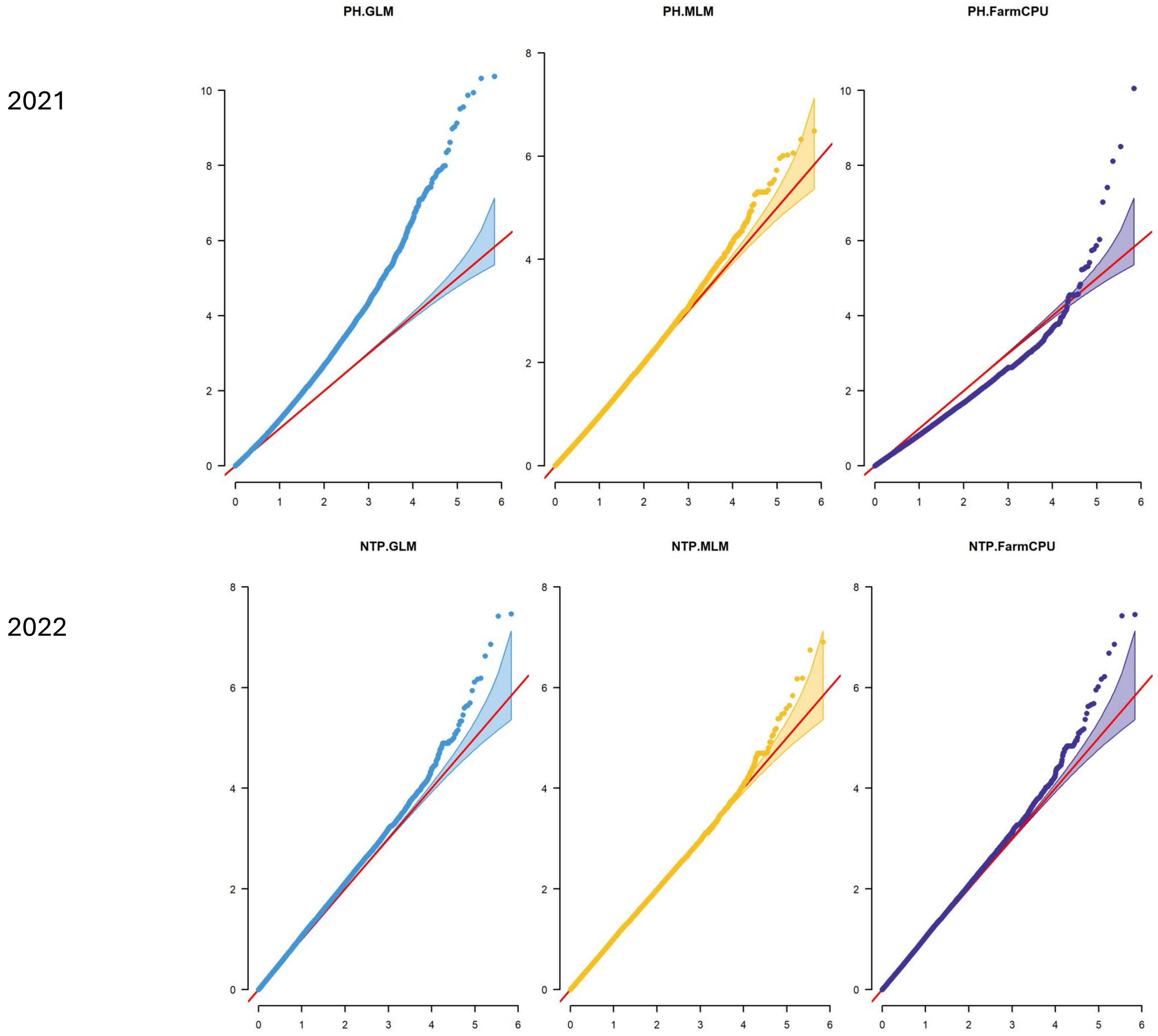

PL

2021

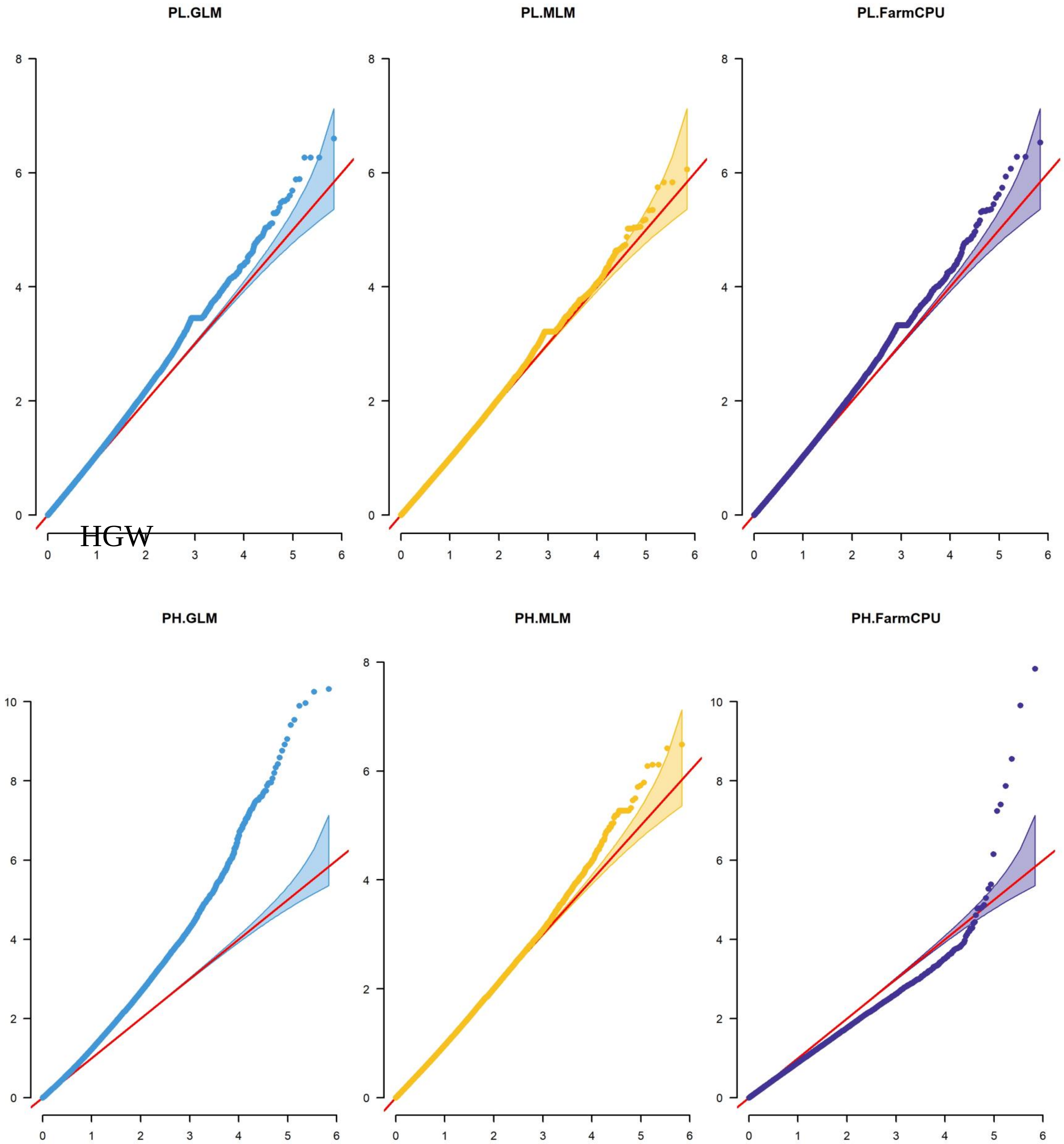

HGW

2022

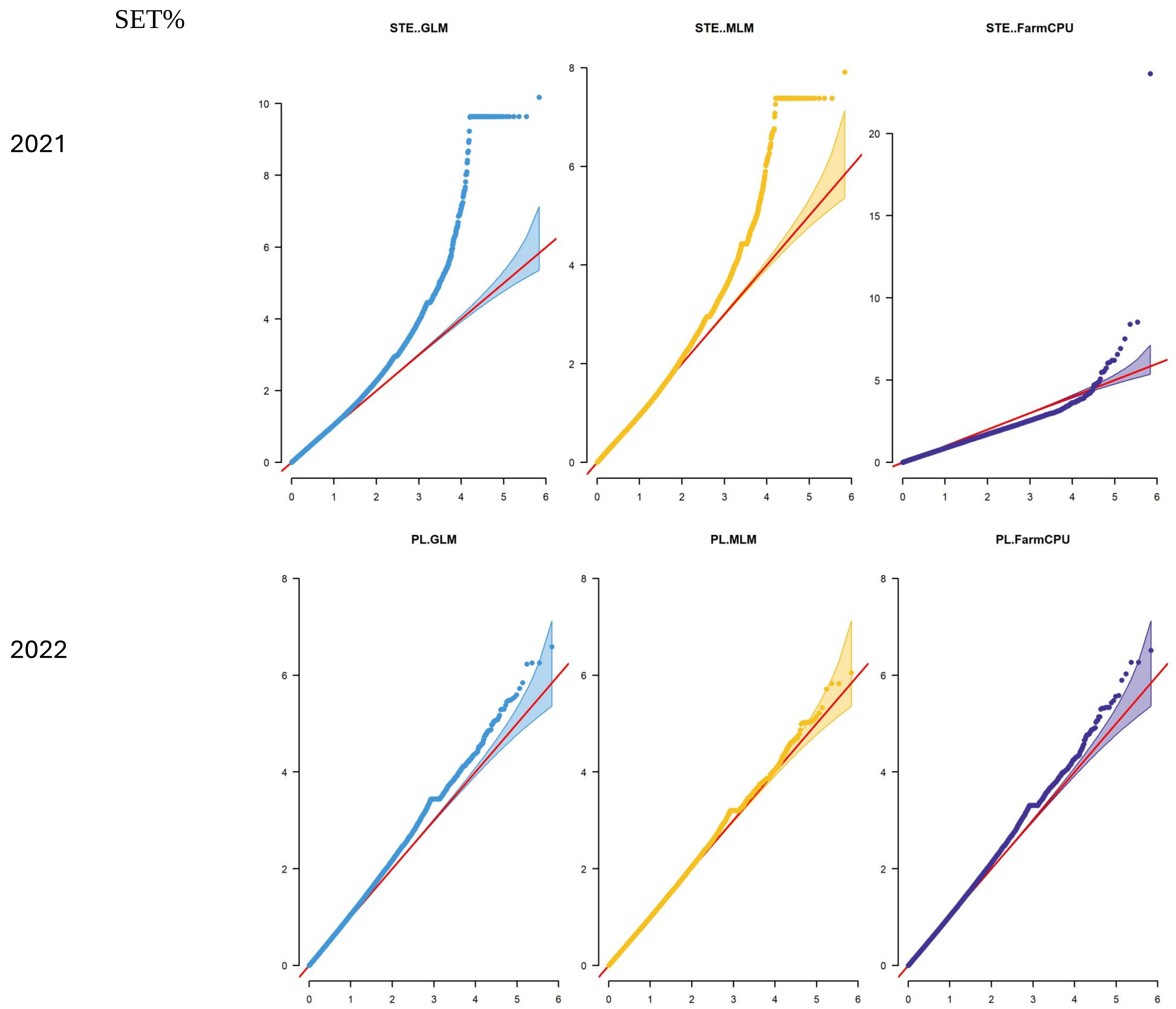
SET%
2021
2022
STE..GLM
STE..MLM
STE..FarmCPU
PL.GLM
PL.MLM
PL.FarmCPU

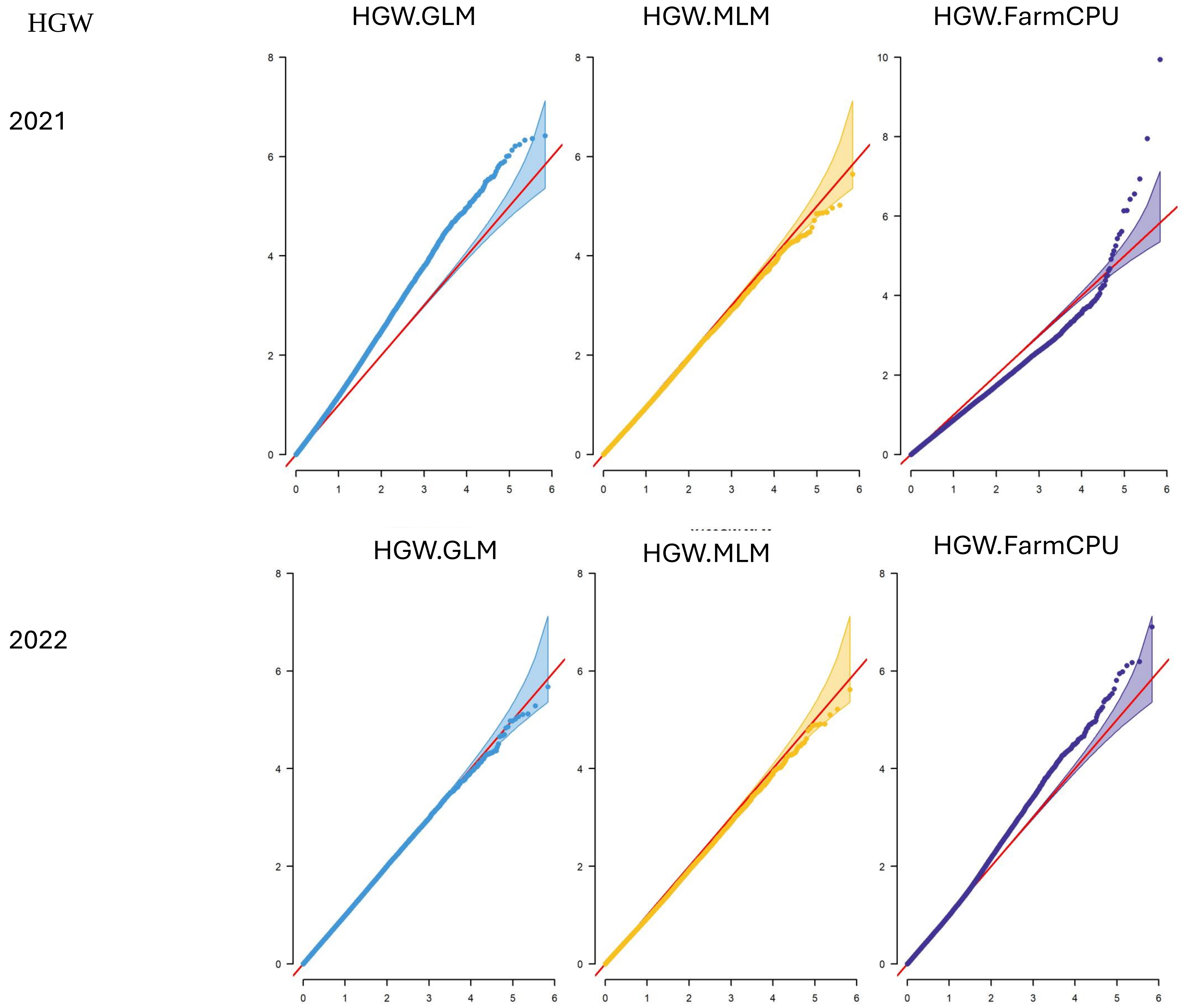

Supplementary Figure2: QQ plots for all traits scored under water deficit condition in the two growing season of 2021 and 2022
