# Supplementary material for: Genome-Wide Association Study of Rice Diversity Panel Reveals New QTLs for Tolerance to Water Deficit Under the Egyptian Conditions
Source: Rice (N Y). 2024 Apr 23;17:29. doi: 10.1186/s12284-024-00703-1 (PMC11035518; doi:10.1186/s12284-024-00703-1)
Supplement: Supplementary file 1 — Additional file 1: Supplementary Figure 1. QQ plots for all traits scored under normal condition in the two growing season of 2021 and 2022. Supplementary Figure 2. QQ plots for all traits scored under water deficit condition in the two growing season of 2021 and 2022. Supplementary Figure 3. The Manhattan plot for all traits scored under FI conditions in the two-growing season of 2020 and 2021. Supplementary Table 1. List of 392 O. sativa accessions and country of origin, principle component structure and fast structure assignment. Supplementary Table 2. the average sum of ranks for all 392 genotypes in each trait at the two growing seasons. Supplementary Table 3. the significant SNPs associated with NDH, FLA, PH, NTP, NPP, PL, HGW and SET% under DI and FI irrigation at the first grwoing season (2021). Supplementary Table 4. the significant SNPs associated with NDH, FLA, PH, NTP, NPP, PL, HGW and SET% under DI and FI irrigation at the second grwoing season (2022). [file 12284_2024_703_MOESM1_ESM.zip › Supplementary Figure1.pdf]

FLA

2021

2022

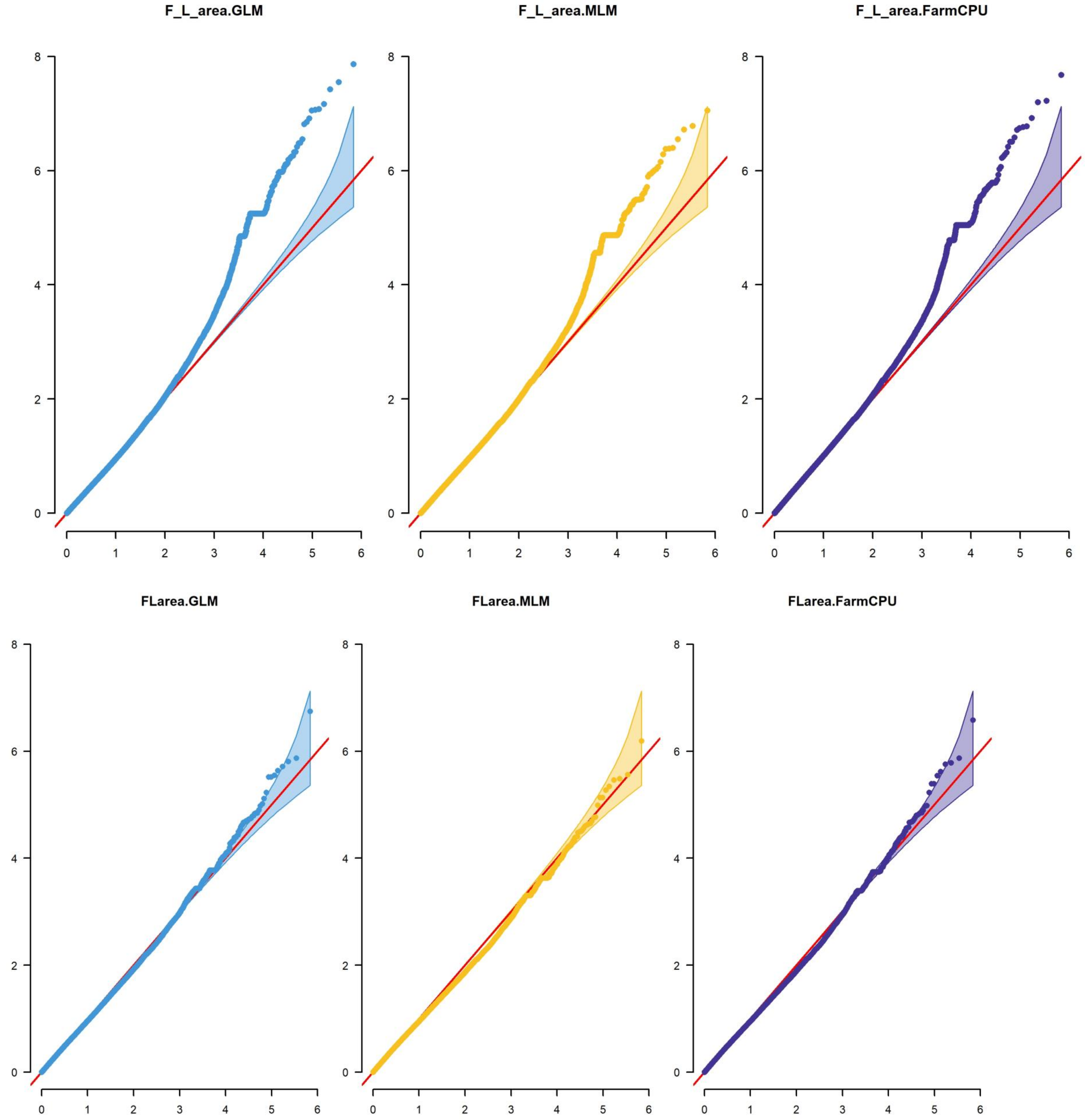

Supplementary Figure1: QQ plots for all traits scored under normal condition in the two growing season of 2021 and 2022

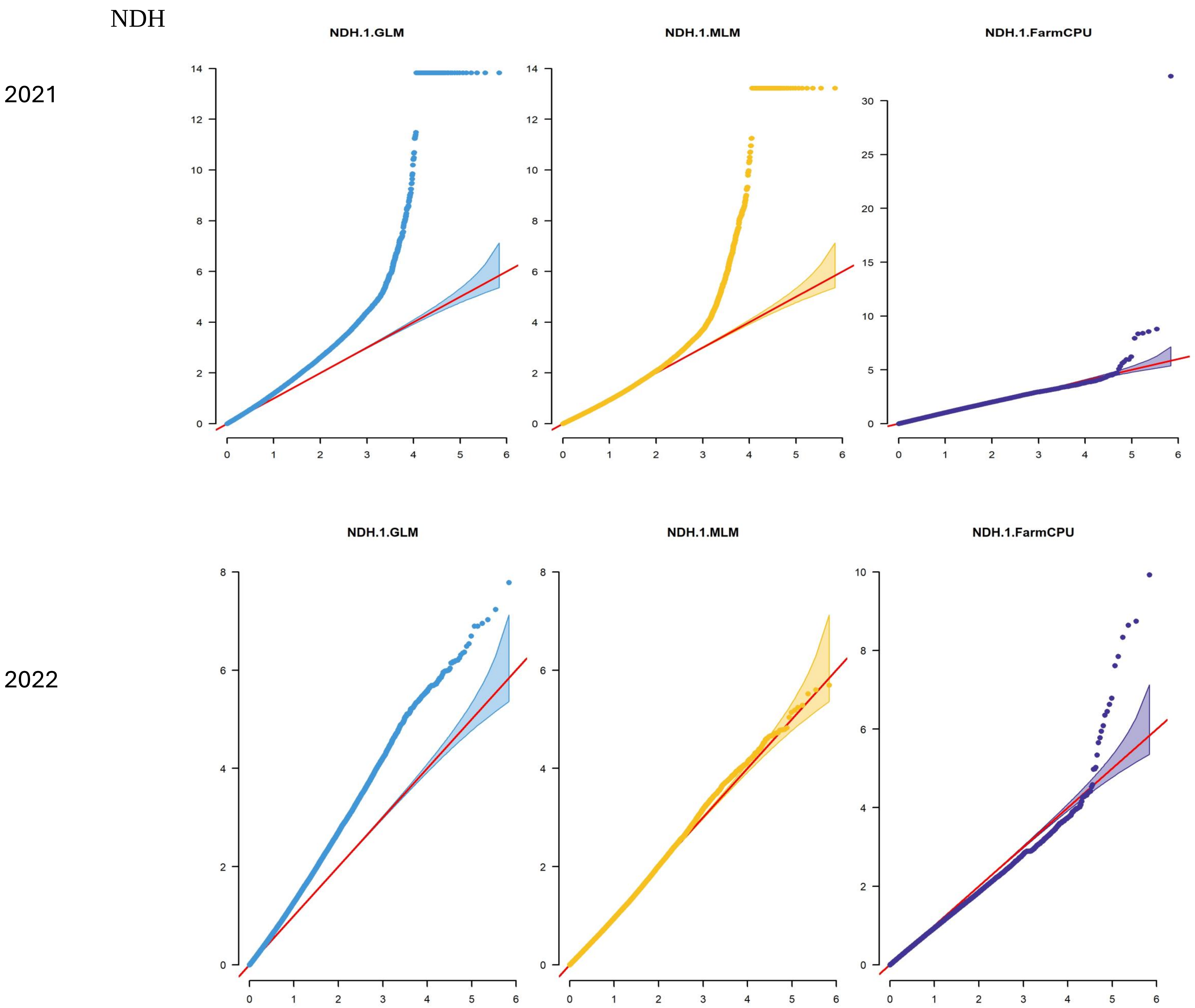
NDH
2021
2022
NDH.1.GLM
NDH.1.MLM
NDH.1.FarmCPU
NDH.1.GLM
NDH.1.MLM
NDH.1.FarmCPU
0
1
2
3
4
5
6
0
2
4
6
8
10
12
14
0
5
10
15
20
25
30
0
2
4
6
8
10

NPP

2021

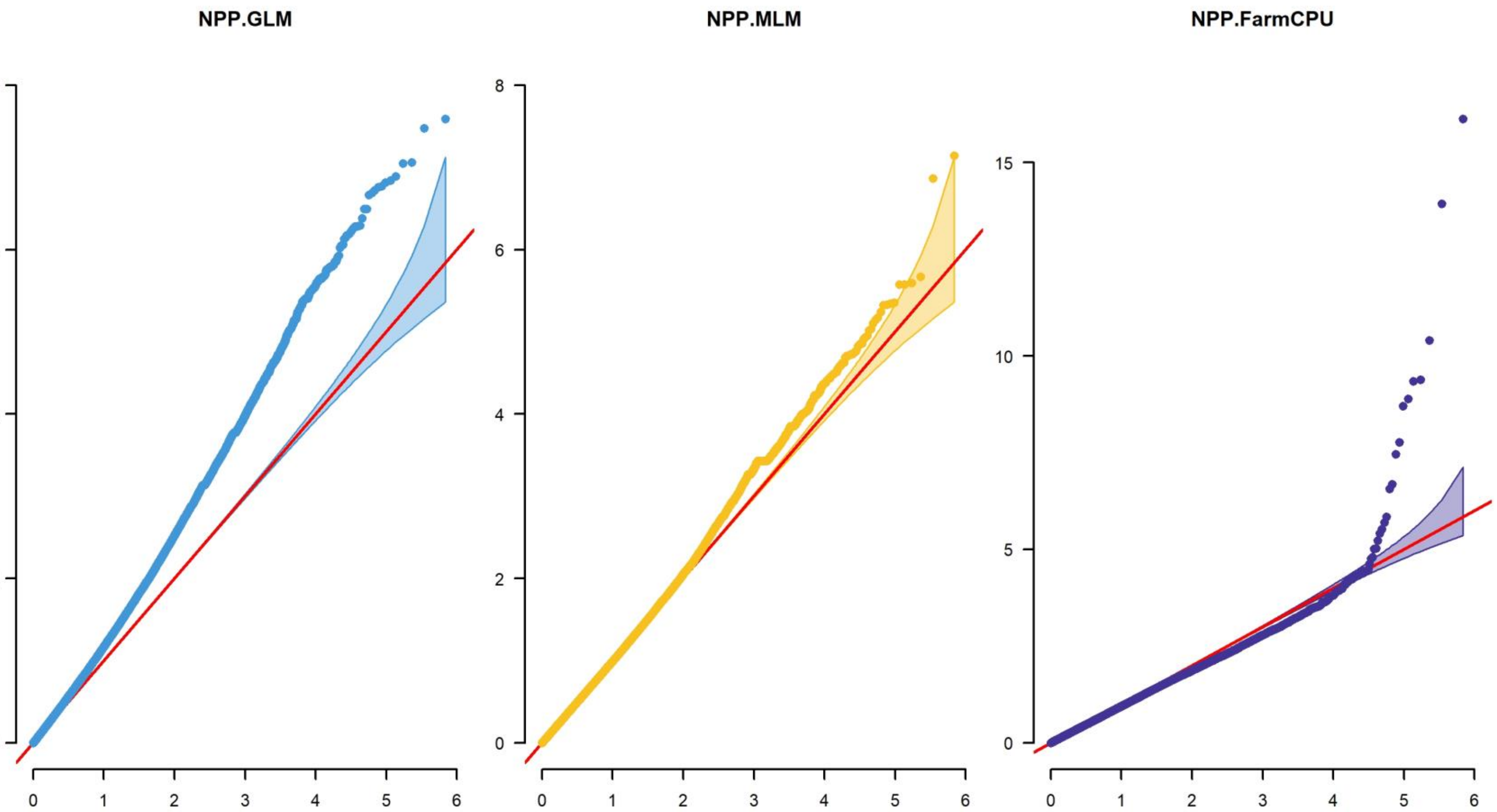

2022

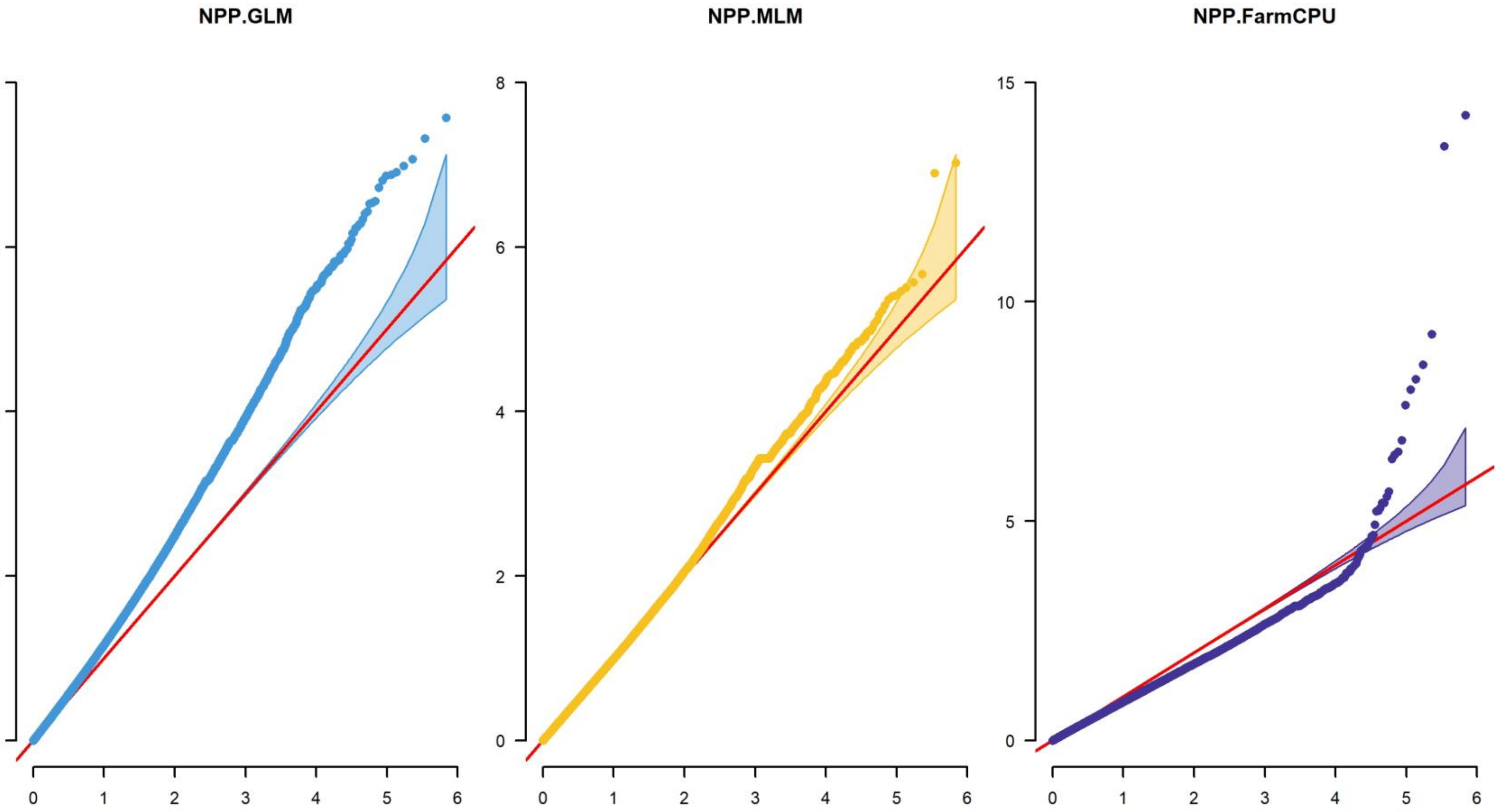

NTP

2021

2022

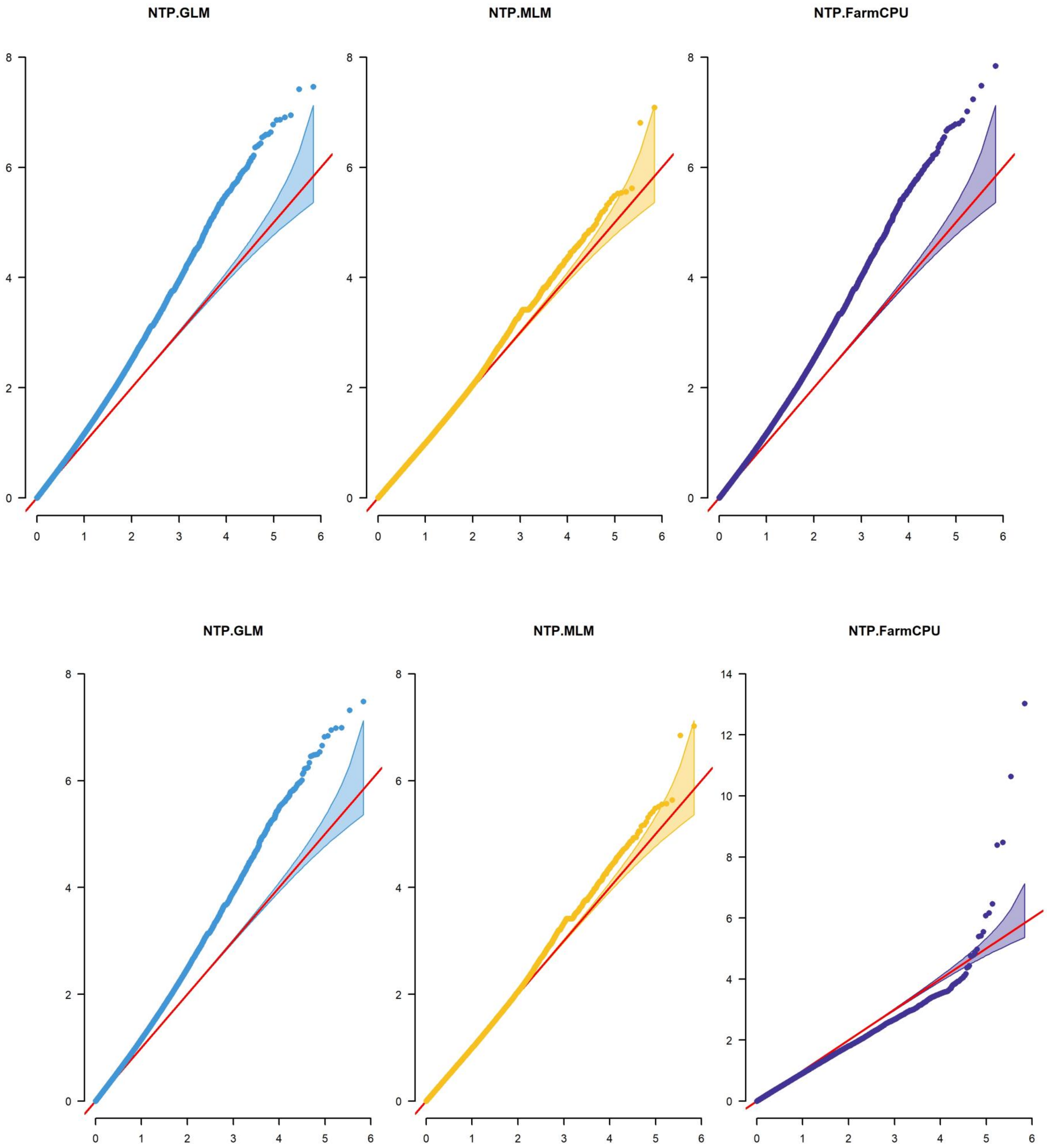

PH

2021

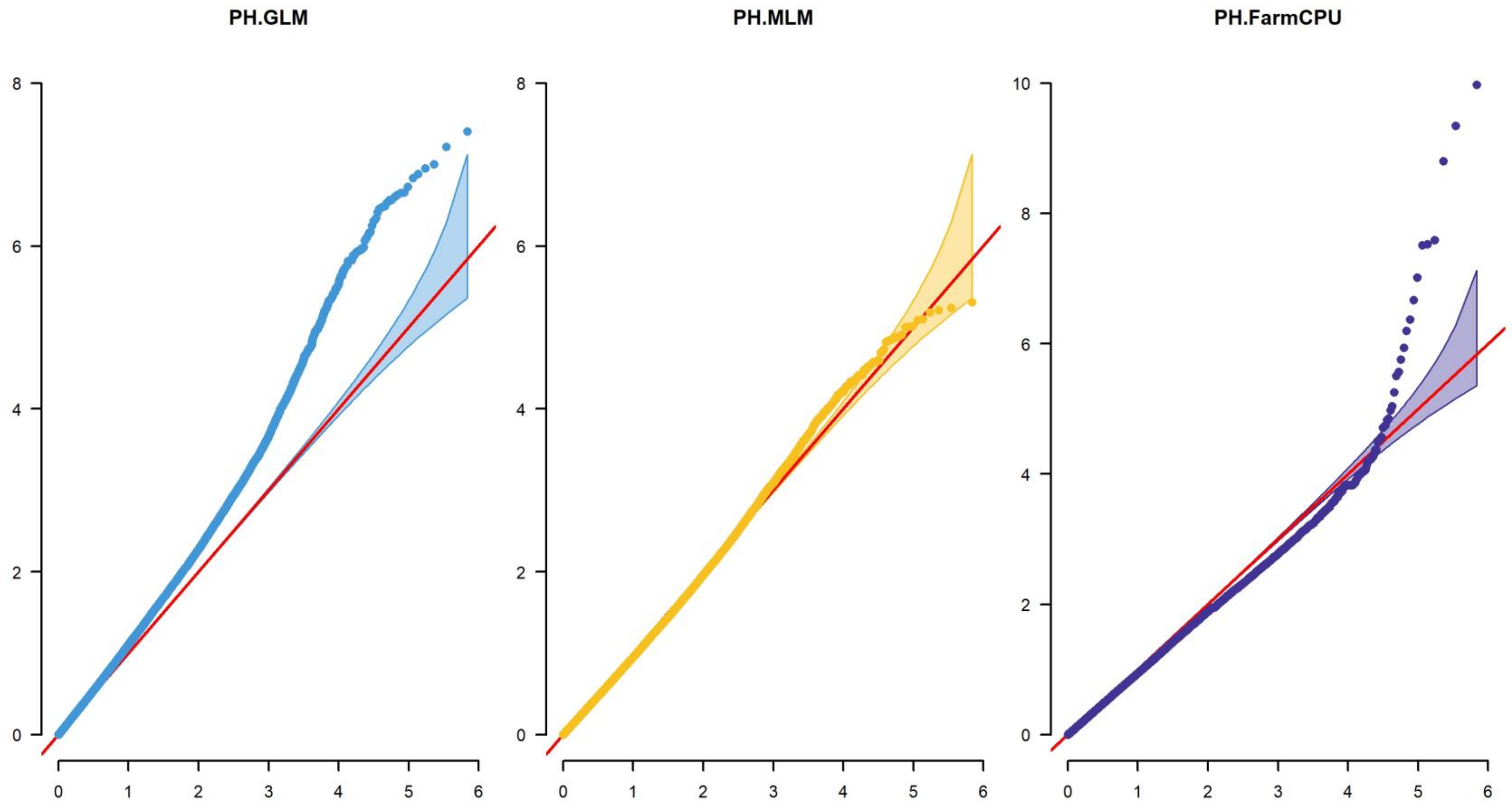

2022

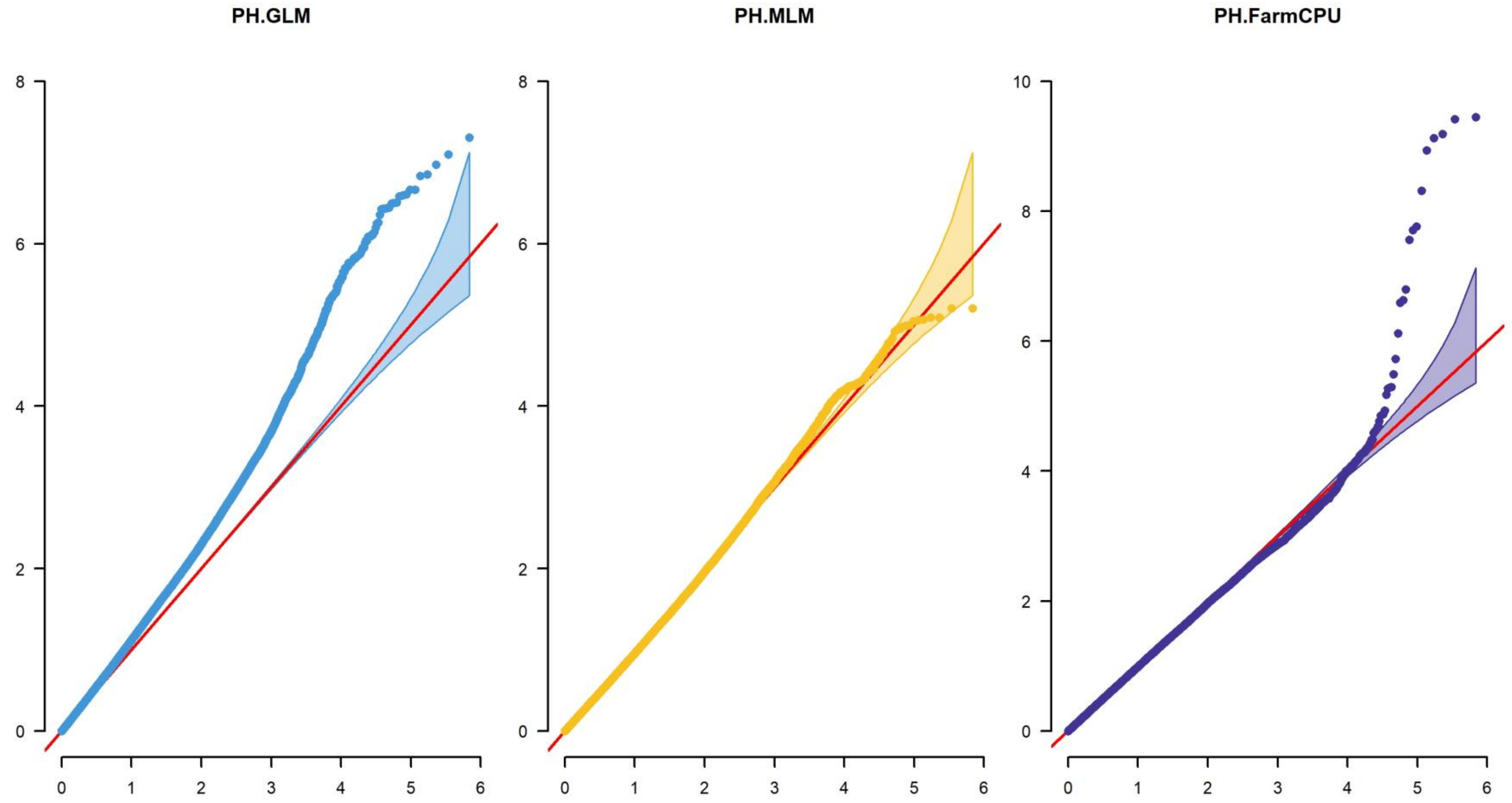

PL

2021

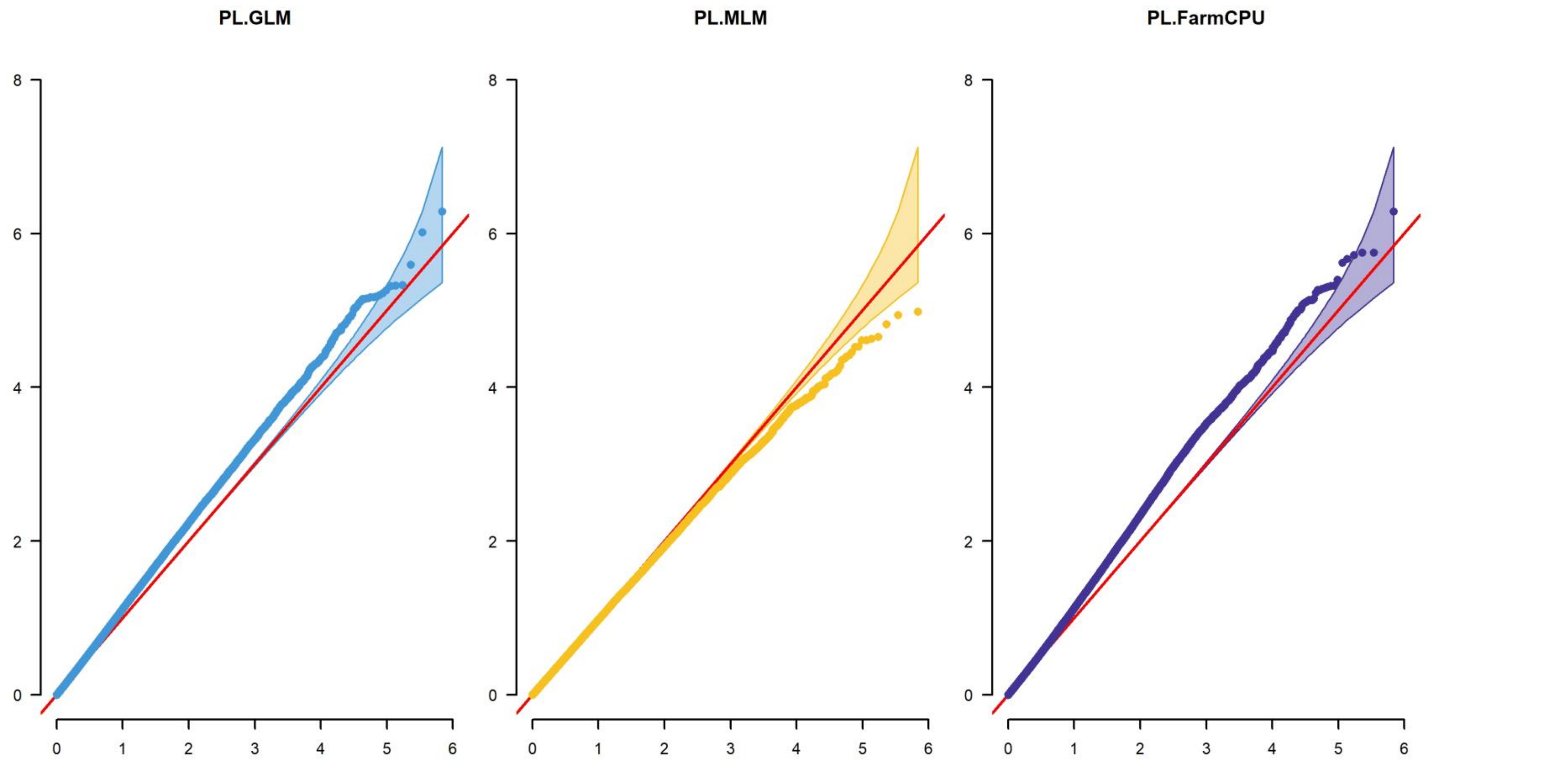
PL.GLM
PL.MLM
PL.FarmCPU
0 2 4 6 8
0 1 2 3 4 5 6

2022

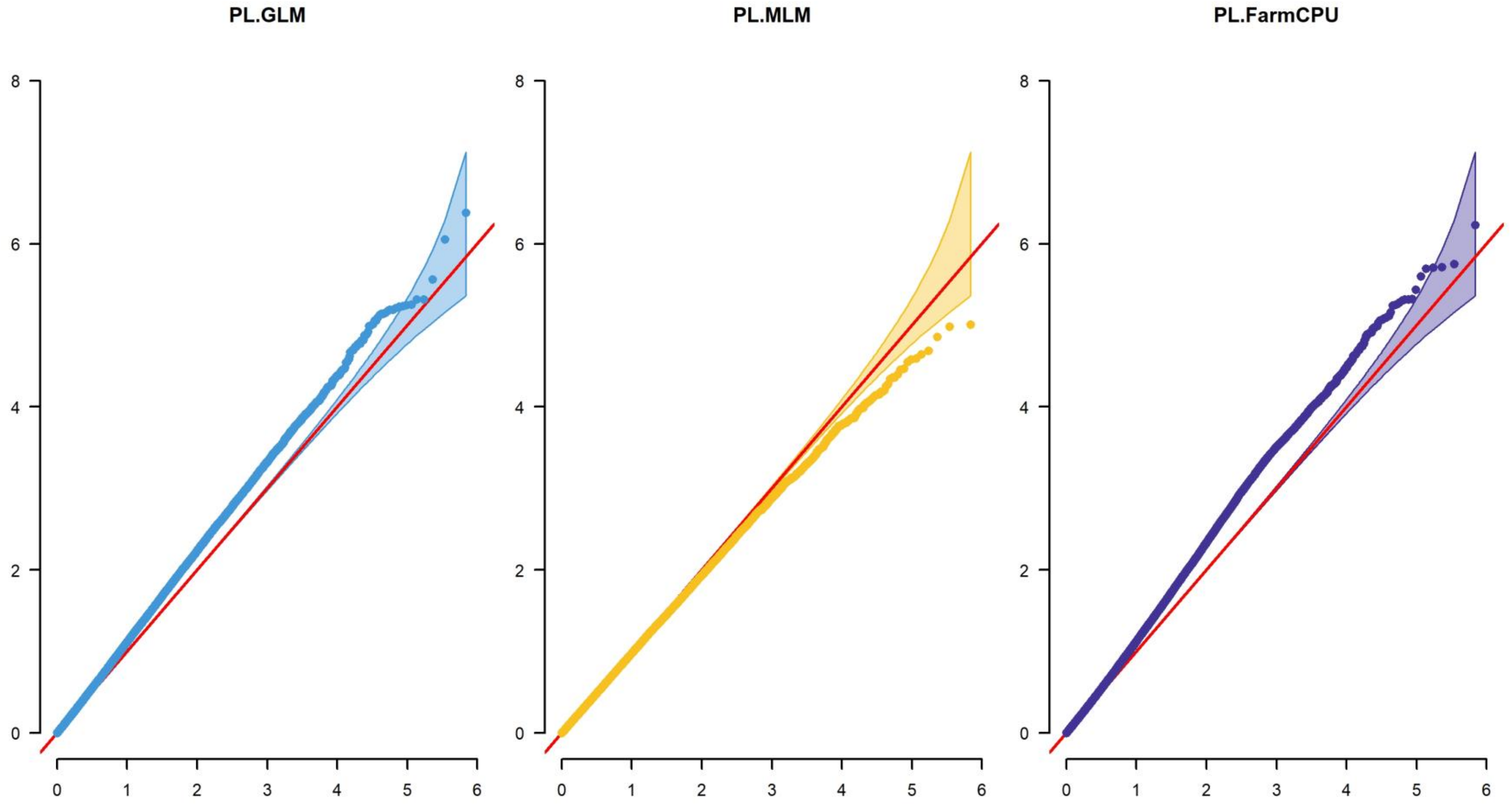
PL.GLM
PL.MLM
PL.FarmCPU
0 2 4 6 8
0 1 2 3 4 5 6

SET%

2021

2022

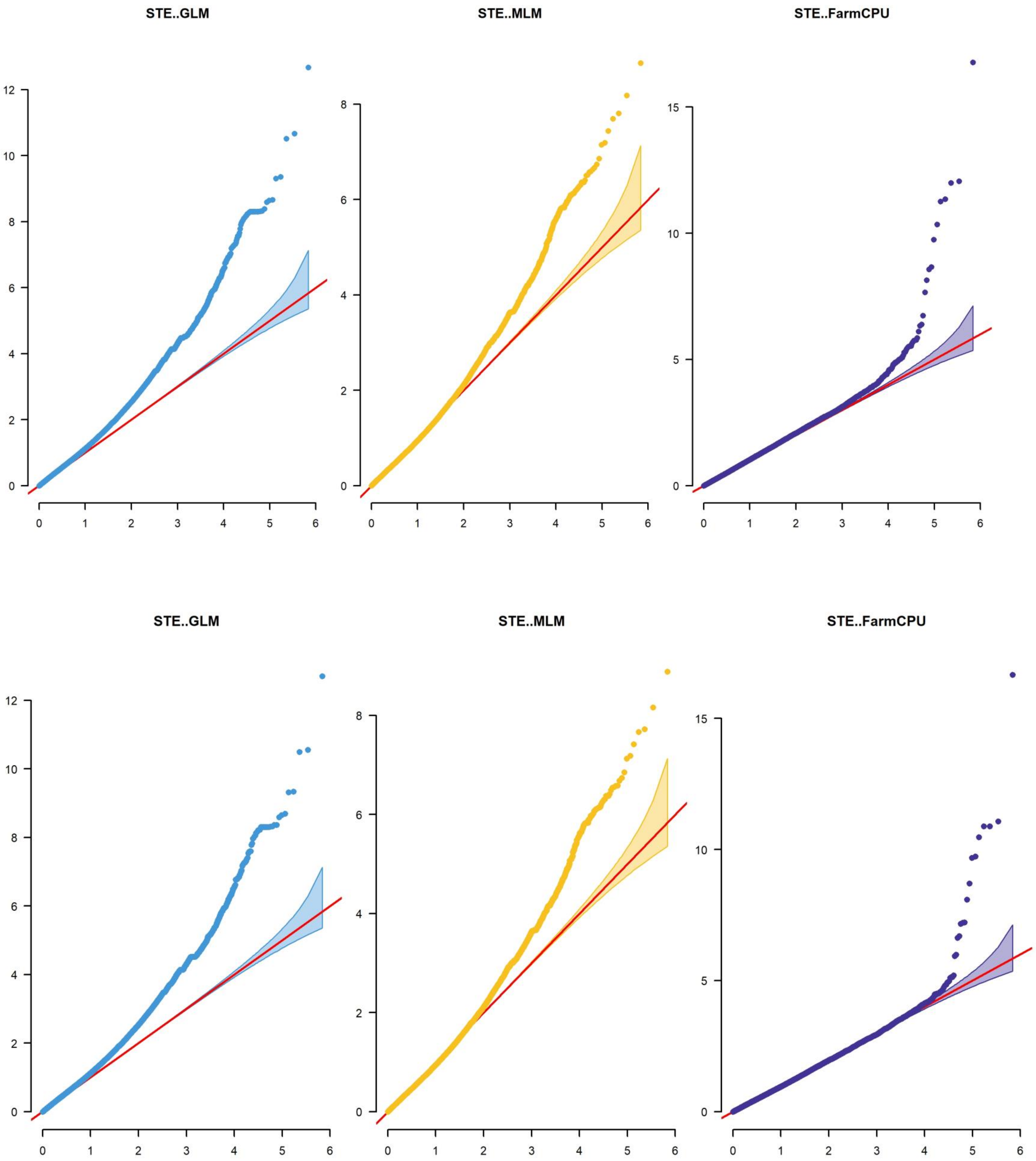

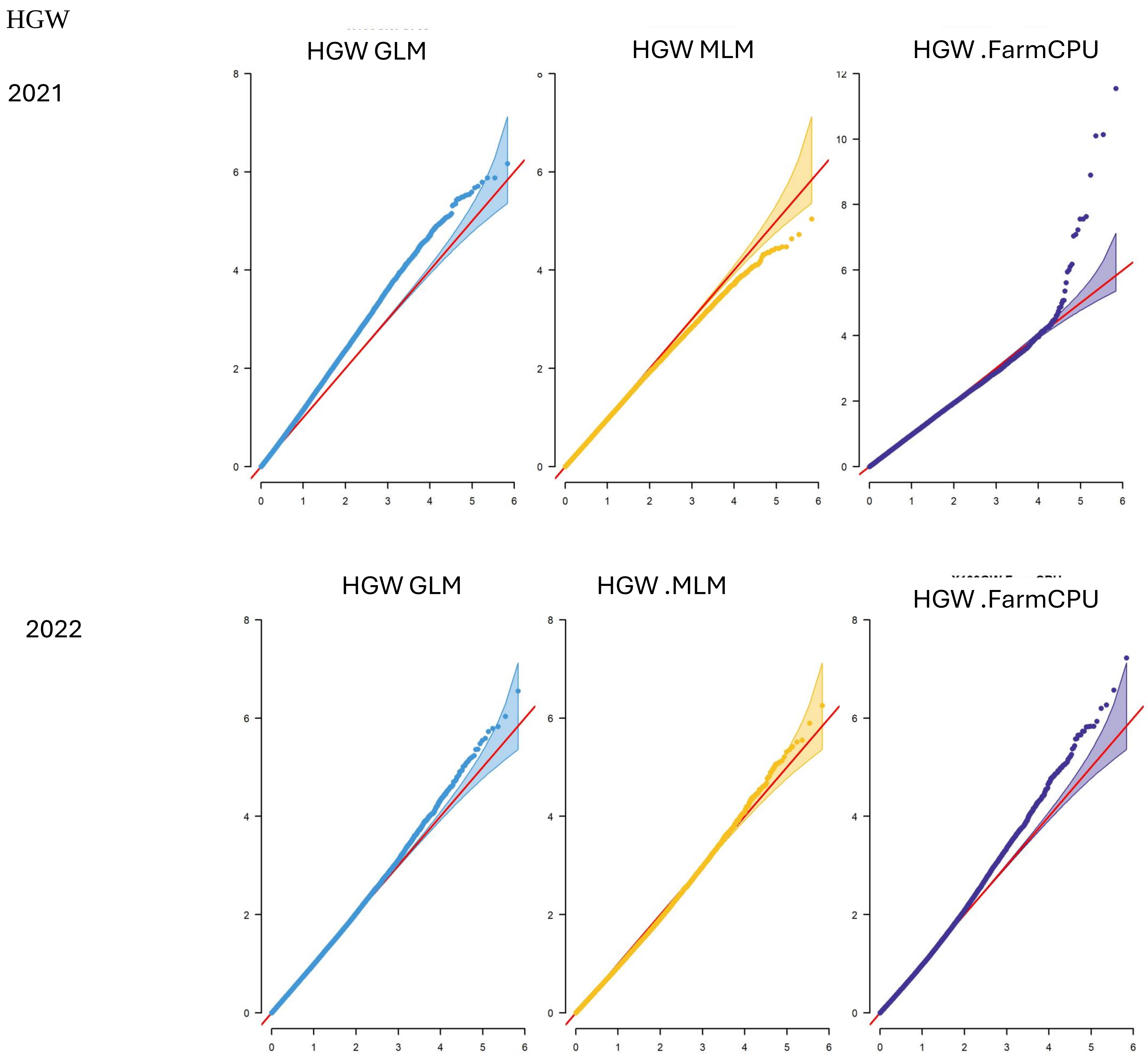

Supplementary Figure1: QQ plots for all traits scored under normal condition in the two growing season of 2021 and 2022
