# Supplementary material for: Genome-Wide Association Study of Rice Diversity Panel Reveals New QTLs for Tolerance to Water Deficit Under the Egyptian Conditions
Source: Rice (N Y). 2024 Apr 23;17:29. doi: 10.1186/s12284-024-00703-1 (PMC11035518; doi:10.1186/s12284-024-00703-1)
Supplement: Supplementary file 1 — Additional file 1: Supplementary Figure 1. QQ plots for all traits scored under normal condition in the two growing season of 2021 and 2022. Supplementary Figure 2. QQ plots for all traits scored under water deficit condition in the two growing season of 2021 and 2022. Supplementary Figure 3. The Manhattan plot for all traits scored under FI conditions in the two-growing season of 2020 and 2021. Supplementary Table 1. List of 392 O. sativa accessions and country of origin, principle component structure and fast structure assignment. Supplementary Table 2. the average sum of ranks for all 392 genotypes in each trait at the two growing seasons. Supplementary Table 3. the significant SNPs associated with NDH, FLA, PH, NTP, NPP, PL, HGW and SET% under DI and FI irrigation at the first grwoing season (2021). Supplementary Table 4. the significant SNPs associated with NDH, FLA, PH, NTP, NPP, PL, HGW and SET% under DI and FI irrigation at the second grwoing season (2022). [file 12284_2024_703_MOESM1_ESM.zip › Supplementary Figure 3.pdf]

NDH-N2021

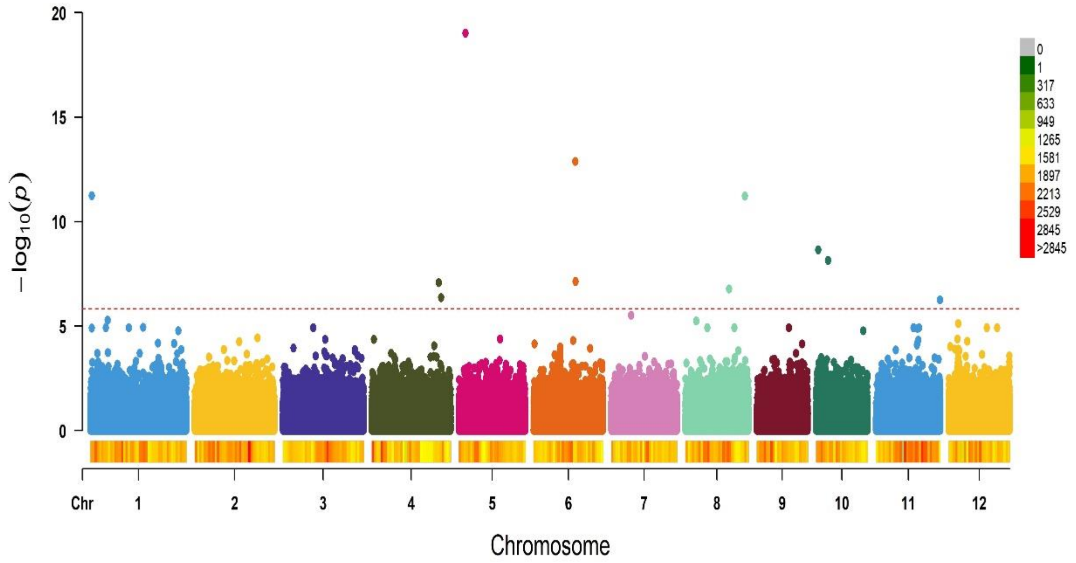

NDH-N2022

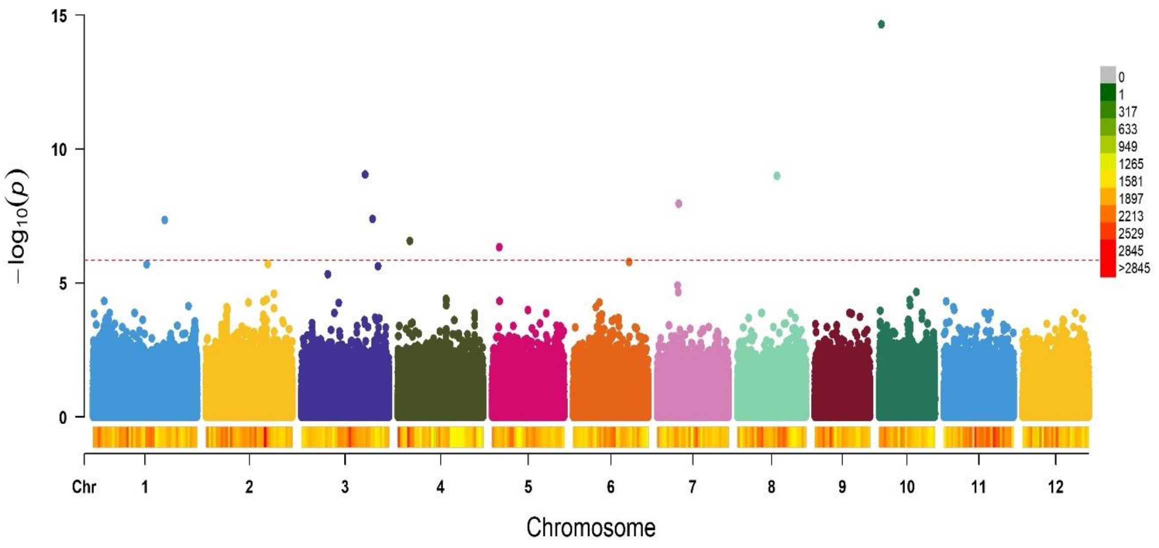

FLA-N2021

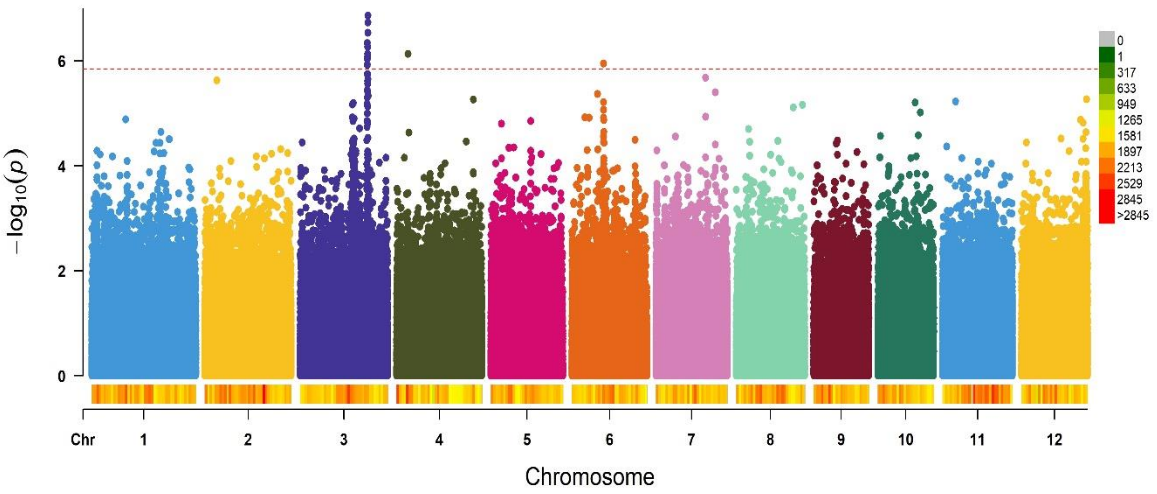

FLA-N2022

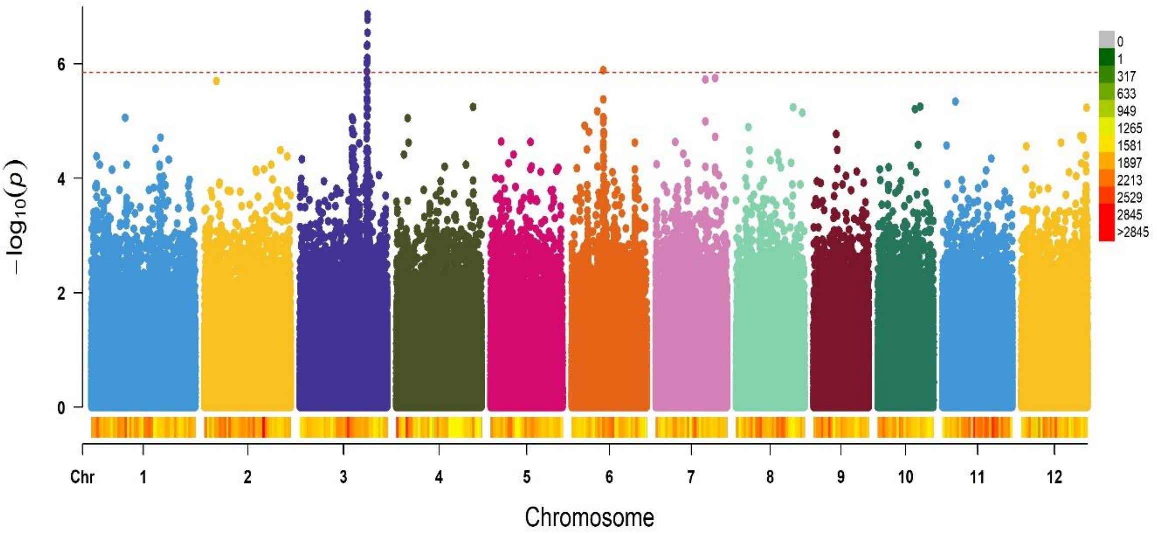

PH-N2021

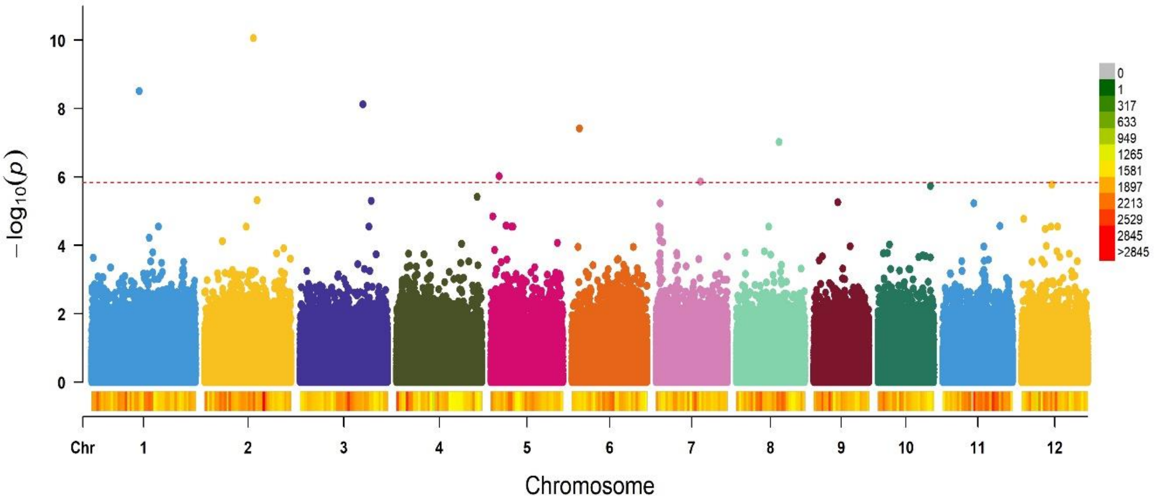

PH-N2022

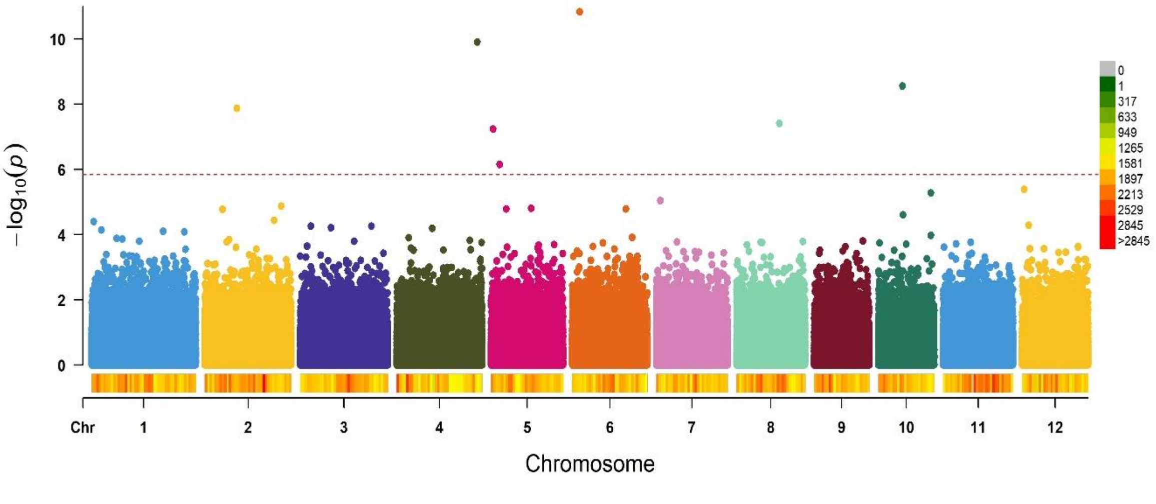

NTP-N2021

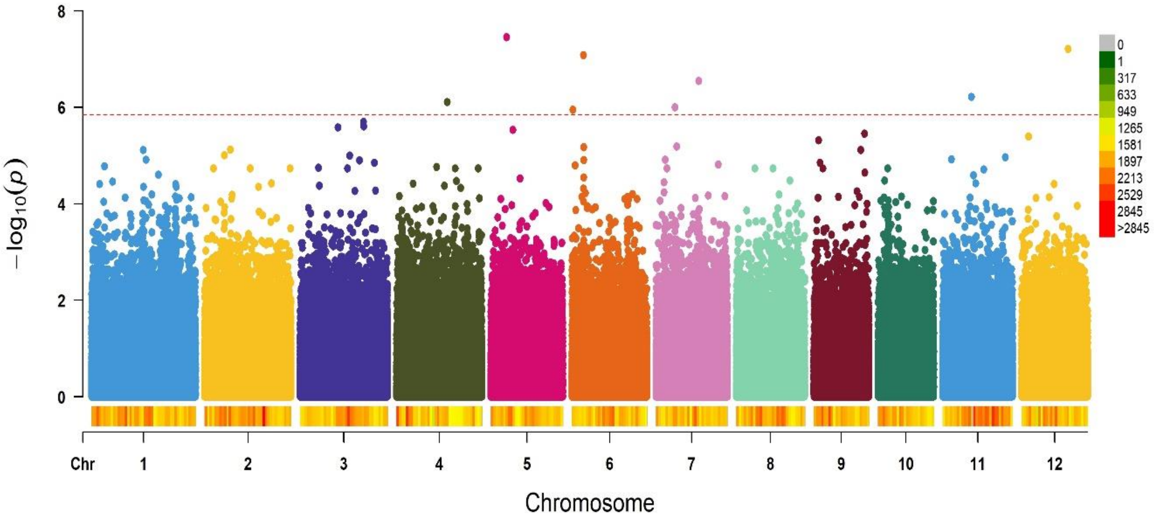

NTP-N2022

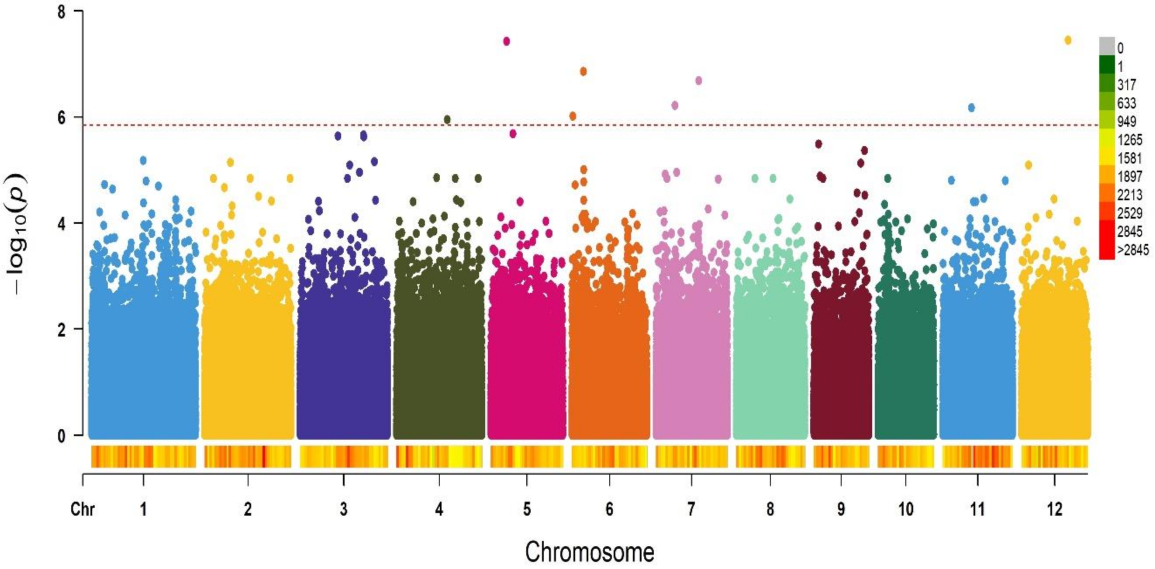

NPP-N2021

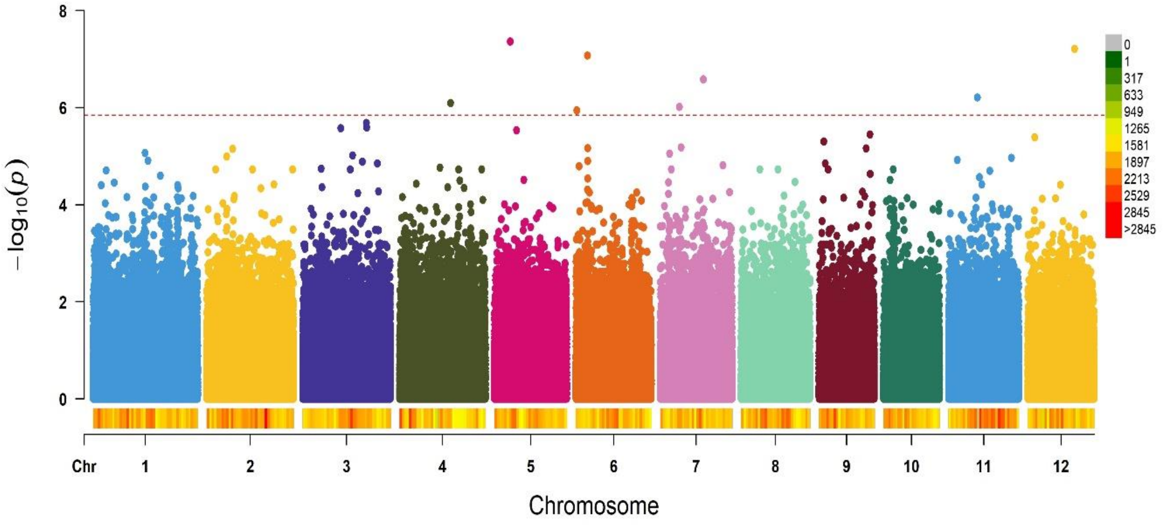

NPP-N2022

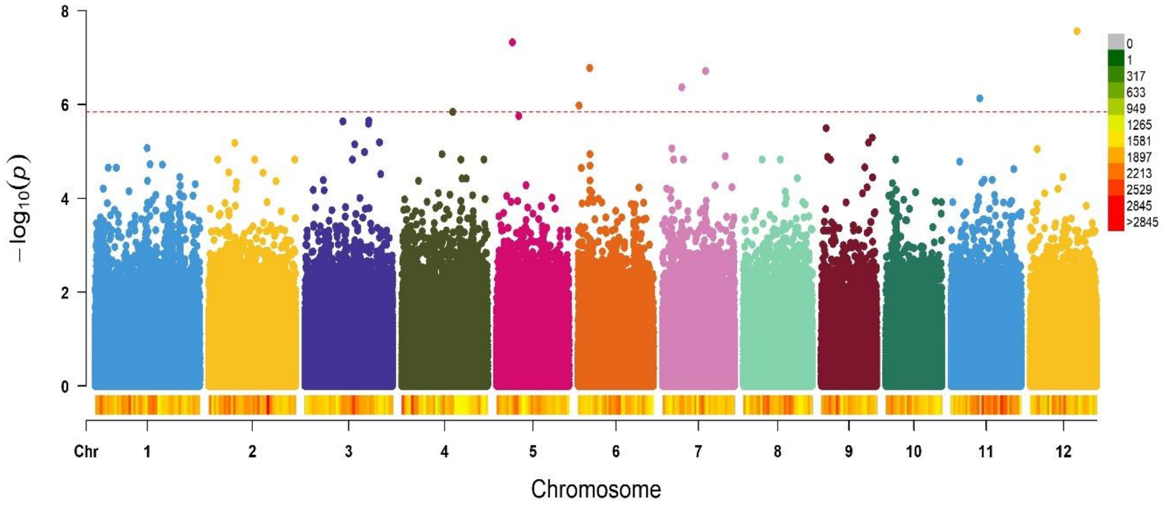

PL-N2021

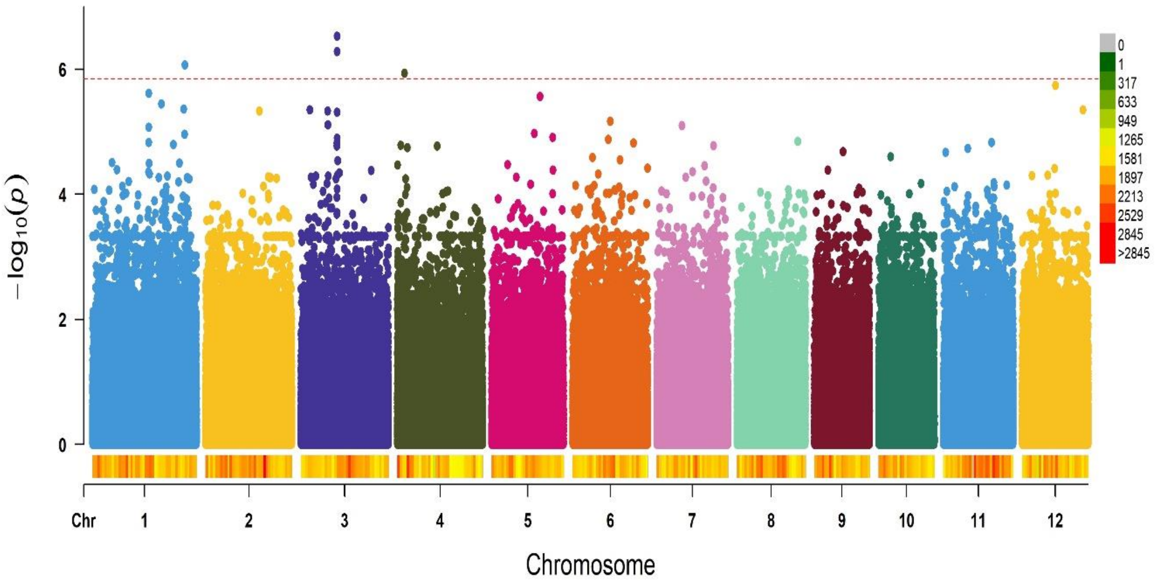

PL-N2022

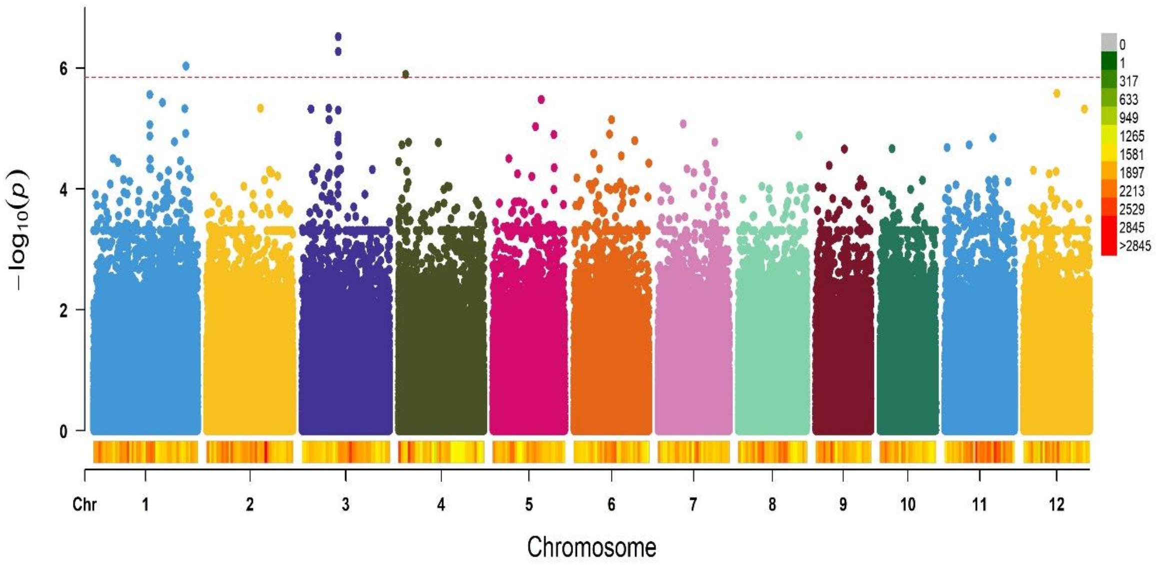

HGW-N2021

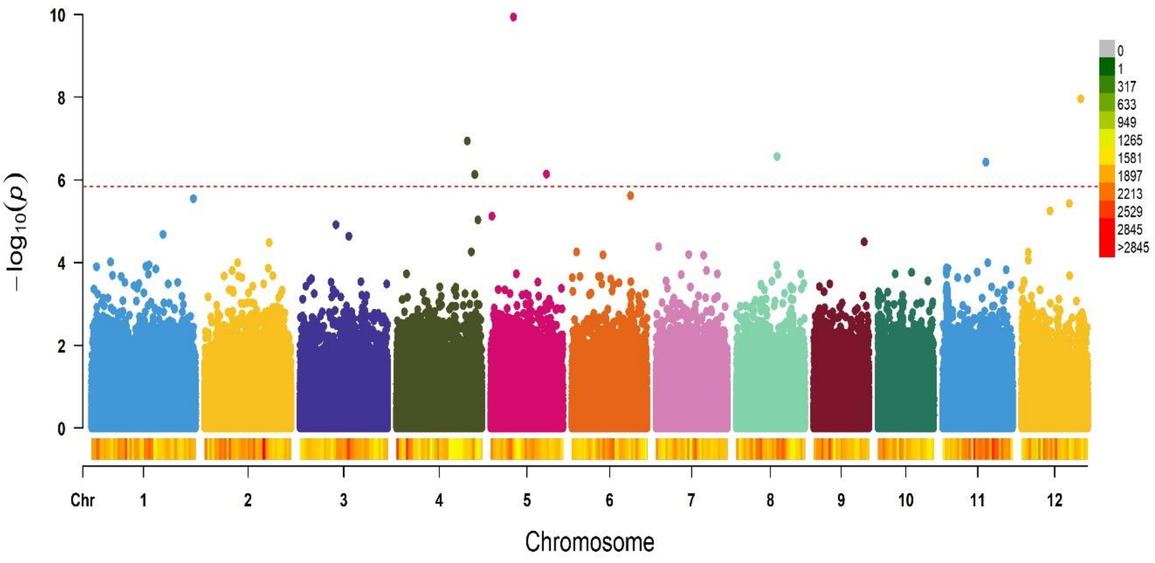

HGW-N2022

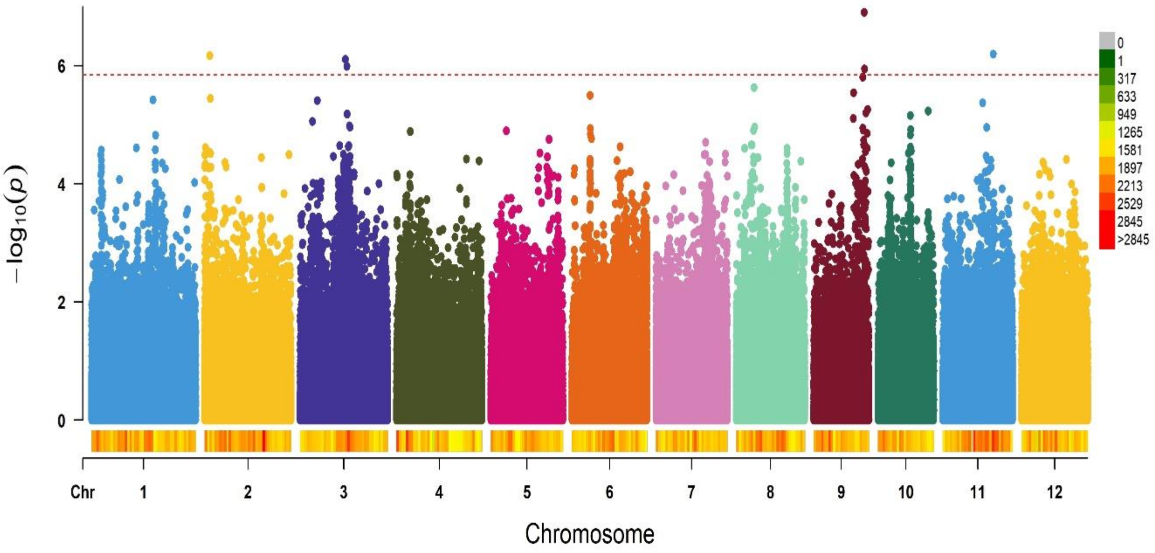

SET%-N2021

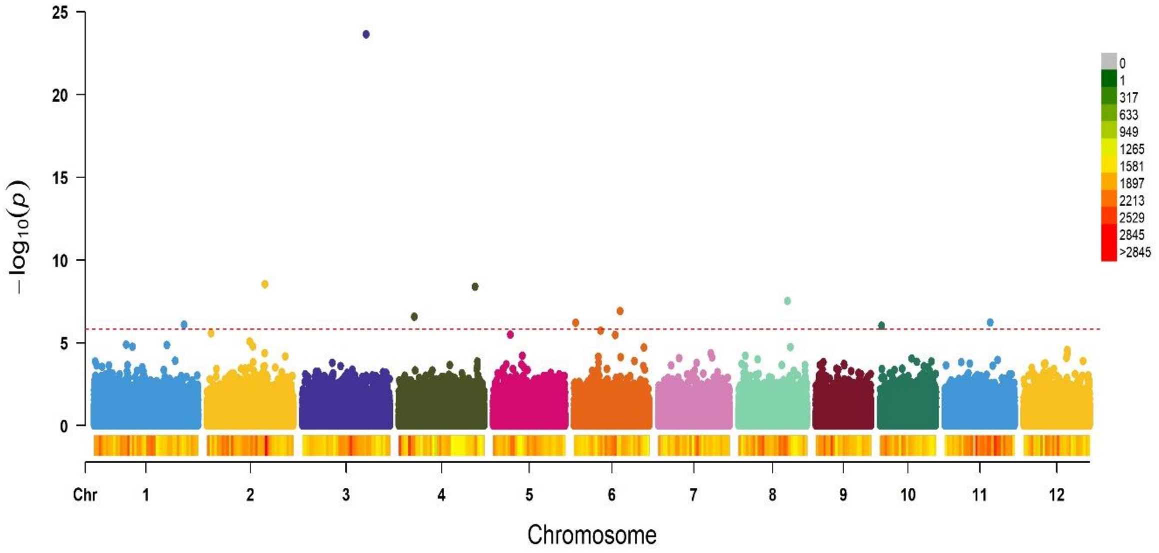

SET%-N2022

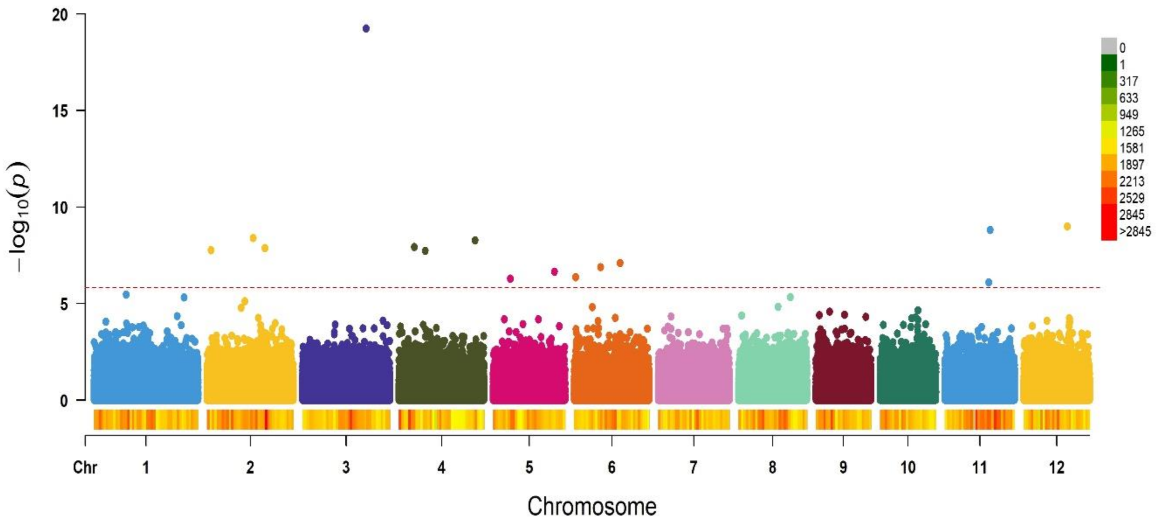

Supplementary Figure 3: The Manhattan plot for all traits scored under FI conditions in the two-growing season of 2020 and 2021.
